# Machine learning-driven decision support for antibiotic optimization in typhoid fever based on patient profiles

**DOI:** 10.1186/s12911-026-03528-8

**Published:** 2026-05-12

**Authors:** Charles Ssemuyiga, Elminah Saru, Yusuf Abbas Aleshinloye

**Affiliations:** 1 PharmaQsar Bioinformatics Firm, Kampala, Uganda; 2 Department of Public Health, School of Public Health, Kampala International University, Kampala, Uganda; 3 Department of Biochemistry and Biotechnology, Pwani University, Kilifi, Kenya; 4 School of Mathematics and Computing, Kampala International University, Kampala, Uganda

**Keywords:** Typhoid fever, Machine learning, Predictive modelling, Precision medicine, Disease triage

## Abstract

**Introduction:**

Typhoid fever remains a major Global public health concern, with treatment outcomes strongly influenced by antimicrobial resistance (AMR) and inter-patient variability. Determining the most appropriate antibiotic for an individual patient remains clinically challenging. Machine learning–based clinical decision support systems (CDSS) offer a promising avenue for improving diagnostic precision and guiding antibiotic selection using routinely collected clinical data.

**Methods:**

We developed a machine learning-based decision-support framework using XGBoost models to predict (i) treatment outcome (binary), (ii) suspected typhoid classification, and (iii) a resistance-proxy score from clinical and engineered features. Model performance was evaluated using AUROC for classification tasks and R^2^ for regression, alongside probability calibration analysis using the Brier score. SHAP was used to interpret feature importance, generate patient-level explanations, and identify latent patient subgroups. A counterfactual drug-simulation experiment was further implemented to compare clinician-prescribed antibiotics with model-recommended alternatives.

**Results:**

The treatment outcome classifier demonstrated strong generalization performance, achieving a test AUROC of 0.962 ± 0.010 and an overall accuracy of 90%. The suspected typhoid classifier achieved an AUROC of 0.902 ± 0.005 with an overall classification accuracy of 82%. The resistance-proxy regression model showed moderate predictive capacity (R^2^ = 0.588 ± 0.011). SHAP analysis identified platelet count, age, hemoglobin, calcium, potassium, and severity score as dominant predictors across models and revealed biologically coherent patient subgroups through attribution-based clustering. Counterfactual drug simulations showed that the model’s top recommendation matched the clinician-prescribed drug in 37.1% of cases and appeared as the second-rankedt option in 28.2% of cases. Treatment success was highest when prescriptions aligned with the model’s primary recommendation (72.7%) and lowest when no alignment was observed (32.6%).

**Conclusion:**

This study demonstrates the feasibility of using machine learning to simulate antibiotic selection in typhoid treatment using patient-level clinical profiles. It presents a machine learning-based decision-support framework for antibiotic optimization under uncertainty, with explicit relevance to antimicrobial resistance management in resource-limited settings. To our knowledge, this is among the first studies to integrate explainable machine learning with counterfactual drug simulation for antibiotic optimization in typhoid fever.

**Supplementary Information:**

The online version contains supplementary material available at 10.1186/s12911-026-03528-8.

## Background

Enteric fever, a systemic bacterial disease caused by *Salmonella enterica* serovars Typhi and Paratyphi (A, B, and C) collectively referred to as typhoidal Salmonella, remains a significant global health concern [1]. Although the bulk of morbidity and mortality arises from *Salmonella enterica serovar Typhi/S. Typhi* (typhoid fever), infections due to *S. Paratyphi* serovars, which together account for approximately one-fifth of enteric fever cases, are emerging as a notable contributor, especially in regions transitioning to typhoid conjugate vaccine programs [1, 2]. In this study, we focus on typhoid fever. *S. Typhi* is a rod-shaped Gram-negative bacterium of the family *Enterobacteriaceae*. Unlike most *Salmonella* species, *S. Typhi* is a human-restricted pathogen, and the clinical syndrome it produces often mimics other acute febrile illnesses, complicating timely diagnosis and management [2]. The symptoms of typhoid fever usually include a protracted fever, headache, malaise, diarrhea or constipation, stomach pain, and, occasionally, hepatosplenomegaly. Hematological abnormalities like anemia and thrombocytopenia, as well as biochemical disruptions like changed calcium and potassium levels, can be linked to severe disease and correlate with the severity of the illness and the results of treatment [3, 4]. Traditional bacterial identification methods, including culture-based, biochemical, and serological assays, remain resource-and time-intensive [5]. Even advanced molecular techniques such as PCR involve laborious experimental procedures, collectively delaying accurate diagnosis and timely treatment of infections [6].

Antimicrobial resistance (AMR) continues to compromise the effectiveness of antimicrobial therapy and represents a major global public health challenge [5, 7, 8]. Clinical decision-making in infectious diseases commonly relies on surveillance-informed guidelines, empirical rule-based systems such as MYCIN, or clinician judgment; however, these approaches are limited in their ability to account for patient-level variability and evolving resistance patterns [1]. Effective clinical management and international control efforts are highly threatened by the advent of multidrug-resistant (MDR) and extensively drug-resistant (XDR) *S. Typhi* strains, which have drastically reduced therapeutic options for typhoid fever [4, 9].

Recent advances in artificial intelligence (AI) and machine learning (ML) have enabled clinical decision support systems (CDSS), which provide real-time personalized antimicrobial recommendations based on patient-specific factors such as laboratory results, comorbidities, and pathogen profiles [6, 10]. In addition to supporting treatment decisions, AI methods have been applied in epidemiological surveillance, pathogen identification, antimicrobial susceptibility prediction, genome analysis, drug discovery, and vaccine research [11]. These approaches have been associated with improvements in decision-making efficiency and alignment of treatment strategies with resistance patterns, as well as reduced reliance on broad-spectrum antibiotics. ML-based CDSS have also demonstrated potential in resistance prediction and antibiotic optimization through integration of heterogeneous clinical datasets [12, 13].

ML models further enable counterfactual analysis of prescribing decisions by simulating treatment outcomes under alternative drug selections. Such approaches have been applied using interpretable clustering and fuzzy logic to guide ICU medication decisions and reduce unnecessary antibiotic use in primary care while maintaining efficacy [14, 15]. Additionally, AI-driven decision support systems such as HIV-TRePS demonstrate strong predictive performance across diverse patient populations, highlighting the translational potential of probabilistic models in infectious disease management and antimicrobial stewardship [16].

Advances in explainable artificial intelligence (XAI) have improved the interpretability of machine learning models, facilitating their use in clinical settings. Unlike traditional “black-box” approaches, XAI methods provide insight into how input variables contribute to model predictions. These techniques have been applied in the diagnosis and management of infectious and febrile illnesses, where clinical presentations are often heterogeneous and complex [17–19]. For example, explainable models have been used in disease classification, surveillance, and decision support, enabling visualization of feature contributions and supporting clinical interpretation of model outputs [19, 20]. This is particularly relevant in resource-limited settings, where interpretability is important for adoption. However, most XAI applications have focused on disease classification rather than treatment optimization. This study addresses this gap by incorporating SHAP-based interpretability into a machine learning framework for antibiotic selection and treatment outcome prediction in typhoid fever.

We applied Extreme Gradient Boosting (XGBoost), an ensemble learning method based on gradient-boosted decision trees, to develop predictive models for three tasks: resistance proxy score regression, classification of suspected typhoid fever, and treatment outcome prediction. XGBoost is well-suited to clinical datasets due to its ability to handle missing data, class imbalance, and heterogeneous features, while maintaining computational efficiency [12, 21]. The method incorporates second-order gradient optimization, regularization, and sparse data handling, as well as parallelization and feature subsampling to improve performance [21]. These characteristics support its use in biomedical applications involving complex, non-linear relationships. Its application in clinical outcome prediction, resistance modeling, and infectious disease analytics has been reported in recent studies, including in resource-limited settings [22–24].

In this study, we developed a machine learning framework using patient biochemical and demographic data to support antibiotic selection in typhoid treatment. XGBoost was implemented for three predictive tasks: (i) treatment outcome classification, (ii) resistance proxy score regression, and (iii) suspected typhoid classification. We further conducted medication simulation experiments to compare observed prescribing decisions with predicted antibiotic success probabilities. This approach demonstrates the feasibility of AI-assisted decision support for typhoid management and its potential application in antimicrobial stewardship and precision medicine in resource-limited settings.

### Clinical and biological significance of dataset parameters

Understanding the clinical relevance of dataset variables is essential for developing models that provide interpretable and clinically meaningful outputs. Hemoglobin, which reflects oxygen-carrying capacity, has normal ranges of 13.5–17.5 g/dL in men and 12.0–15.5 g/dL in women. Reduced levels indicate anemia, which in typhoid may be associated with chronic infection or bone marrow suppression and may influence treatment decisions [25]. Platelets are essential for blood clotting, and abnormal counts may result from bleeding and thrombotic complications. The normal range is 150,000-450,000/μL [26]. Thrombocytopenia, which increases the risk of bleeding, is indicated by counts below 150,000/μL, whereas thrombocytosis, which may indicate inflammation or myeloproliferative diseases, is indicated by levels exceeding 450,000/μL. Typhoid fever frequently causes thrombocytopenia, which can indicate the severity of the illness or its consequences, such as disseminated intravascular coagulation [26]. Blood coagulation, muscular contraction, nerve transmission, and bone health all depend on *calcium*. It typically ranges between 8.5 and 10.2 mg/dL. While levels above 10.2 mg/dL indicate hypercalcemia, which may result in kidney stones, bone discomfort, or neurological symptoms [27], levels below 8.5 mg/dL indicate hypocalcemia, which may induce muscle cramps, cardiac arrhythmias, or seizures [28]. Due to severe infections or gastrointestinal losses, electrolyte abnormalities, including hypocalcemia, can arise in typhoid patients [29]. *Potassium* is essential for preserving cellular activity, especially in muscle and nerve cells. Between 3.5 and 5.0 mmol/L [30] is the typical range. Whereas levels above 5.0 mmol/L indicate hyperkalemia, which can result in potentially fatal cardiac conduction anomalies, levels below 3.5 mmol/L indicate hypokalemia, which causes cardiac arrhythmias, cramping, or muscle weakness. Diarrhea and vomiting in typhoid can lead to hypokalemia, necessitating electrolyte monitoring and replacement [31]. The severity of symptoms reflects disease progression and guides treatment intensity. It can be mild with low-grade fever, malaise; moderate with high fever, abdominal pain; and severe with delirium, intestinal hemorrhage, or perforation. In typhoid, this correlates with disease stage and complications, influencing hospitalization and treatment strategies [32]. Identifying the causative organism confirms the diagnosis and guides antibiotic therapy. *Salmonella enterica serovar Typhi* is the Primary cause of typhoid fever, whereas other Enterobacteriaceae may indicate co-infections or alternative diagnoses. Duration of therapy is determined by disease severity, response to treatment, and presence of complications. Uncomplicated Typhoid may take 7–14 days, while Complicated Cases may require extended therapy. Monitoring treatment duration assesses adherence and effectiveness, influencing outcomes.

## Materials and methods

### Code availability

The source code for all computational work is available through the GitHub repository [https://github.com/SsemuyigaMHC/TyphoidRx] to ensure transparency, reproducibility, and community validation of all procedures.

### Libraries and computational tools

All analyses, simulations, and predictive modeling procedures were implemented in Python (version 3.12) using a modular set of open-source modules designed for scientific computing and machine learning. In order to effectively organize and modify patient-level clinical and biochemical records, Pandas and NumPy were utilized to handle core data processing and numerical calculations [33, 34]. Dimensionality reduction, logistic regression, k-fold cross-validation, principal component analysis (PCA), and a range of classification metrics such as accuracy, precision, recall, F1-score, AUC-ROC, and confusion matrix visualizations were carried out using Scikit-learn [35]. For ensemble modeling, we employed XGBoost, a highly optimized gradient boosting framework that offers fast and scalable tree-based learning, particularly helpful for tabular and unbalanced clinical data [21]. The imbalanced-learn package’s SMOTE (Synthetic Minority Over-sampling Technique) was used to resolve class imbalance. This improved classification robustness by creating synthetic training samples for the minority class [36]. Exploratory visualization of high-dimensional patient profiles was done using Advanced nonlinear embedding methods, including UMAP and t-SNE [37, 38]. For model interpretability, model outputs were divided into feature-level contributions based on cooperative game theory utilizing SHAP (Shapley Additive exPlanations), making it possible to thoroughly justify treatment predictions [39]. Seaborn, matplotlib-venn, and Matplotlib were used to create the visualizations [40, 41]. To manage file systems, persist trained models, ensure reproducibility, and track resource utilization during testing, auxiliary Python modules including os, joblib, random, and psutil were employed. All computations were executed on a Linux-based workstation with sufficient CPU and memory resources to support parallelized model training and simulation.

### Data acquisition and pre-processing

The dataset comprised 5,760 patient records and 13 variables capturing demographic, clinical, laboratory, microbiological, and treatment-related information for individuals evaluated for typhoid fever. Core features included age, sex, hemoglobin, platelet count, calcium, potassium, blood and urine culture results, administered antibiotic, treatment duration, and treatment outcome (Supplementary File 2, Sheet: Typhoid_Data). The dataset was derived from a retrospective single-center clinical cohort reflecting routine care with follow-up of patient outcomes in an endemic setting. A structured preprocessing pipeline was implemented to ensure data quality and analytical consistency. Initial inspection verified data structure, variable types, and internal consistency. Missing values in numerical variables were imputed using median values, while categorical variables were encoded with an “Unknown” category where appropriate [42]. Records with missing outcome variables were excluded from downstream analysis.

To prevent redundancy and information leakage, a two-stage deduplication strategy was applied. First, duplicate entries were removed based on unique patient identifiers. Second, records sharing identical demographic, clinical, laboratory, microbiological, and treatment attributes were identified and collapsed to a single representative instance, ensuring that identical clinical profiles did not span multiple training folds. Physiological plausibility filters were subsequently applied to remove biologically implausible laboratory measurements. Acceptable ranges were defined as follows: hemoglobin (5–20 g/dL), platelet count (20,000–1,000,000 cells/µL), calcium (5–15 mg/dL), and potassium (2–7 mmol/L). These thresholds encompass extreme but clinically plausible values observed in severe infectious and hematological conditions, while excluding erroneous entries arising from data-entry or instrumentation artifacts [43, 44].

Following preprocessing, the final analytical dataset comprised 1,659 unique and physiologically valid patient records. The primary outcome variable (treatment outcome) exhibited moderate class balance, with 903 cases (54.4%) classified as successful and 756 cases (45.6%) as unsuccessful. To mitigate residual imbalance during model development, SMOTE was applied within the training pipeline [36]. Categorical microbiological variables were harmonized using label encoding following imputation. Continuous variables were retained in their native scale, and dimensionality reduction techniques were applied where appropriate in downstream modeling. This preprocessing framework ensured that the dataset used for machine learning was both clinically coherent and statistically robust.

### Feature engineering

The goal of feature engineering in this analysis was to enhance the predictive value of raw clinical data by encoding, transforming, or combining variables into clinically meaningful features. This process aimed to capture latent patterns associated with typhoid severity, treatment response, and biochemical status, in preparation for further machine learning modeling.

In order to create a severity score for further study, the initial classification of symptoms as Low, Moderate, or High was numerically encoded as 1, 2, and 3, respectively. The agreement between bacterial isolates taken from various biological specimens belonging to the same patient is known as culture concordance. In this study, it specifically denotes whether the same pathogen was isolated from both blood and urine cultures. It often signifies a more severe or invasive disease process, systemic dissemination of the same pathogen, compromised immunity, a higher bacterial load, or delayed clearance [3] and has implications in both diagnostics and treatment selection. Concordance was recorded as 1 if they matched and 0 otherwise. *Salmonella enterica* serovar Typhi (*S. Typhi*) is the primary cause of typhoid fever, a systemic illness transmitted via the fecal-oral route. Diagnosis is ideally confirmed by isolating *S. Typhi* from sterile sites such as blood, bone marrow, or urine, though culture sensitivity can be reduced by prior antibiotics or low bacterial load [3, 45]. Detection of *S. Typhi* in either blood or urine cultures confirms active infection and informs antimicrobial therapy. The “*Salmonella Presence*” feature flags records where *S. Typhi* appears in either culture field, capturing cases that culture concordance might miss, especially when present in only one sample. *Culture Type* reflects refined culture concordance across systemic compartments. In typhoid fever, *S. Typhi* typically appears in the blood early and may later be detected in urine. Using preprocessed blood and urine culture fields, we engineered a categorical variable as follows: 0 = No growth (both cultures negative/missing), 1 = Positive blood culture only, 2 = Positive urine culture only, 3 = Concordant growth (same species in both), 4 = Discordant growth (different species). This feature captures inter-site infection dynamics without prioritizing *S. Typhi*, enabling broader characterization of culture agreement. *Sex Code* accounts for biological sex, which influences immune function, drug metabolism, and baseline hemoglobin levels. The dataset encoded gender as 0 = Female and 1 = Male; this was directly mapped to the Sex Code variable. Potential therapeutic effects or interactions are captured by medication features. Binary indicators were created for each administered medicine using one-hot encoding of the *Current Medication* column. The *Intensity Score* is a squared severity score that magnifies differences between mild, moderate, and severe symptoms, enabling stronger weighting for highly symptomatic cases. *Normalized Treatment Duration* adjusts raw treatment length by clinical severity to reflect deviations from expected recovery time. It was calculated by dividing treatment duration by the severity score [46]. *Resistance Proxy Score* is a composite metric engineered to estimate the likelihood of antimicrobial resistance or treatment failure in suspected typhoid fever cases. It integrates three clinically relevant dimensions: microbiological culture discordance, symptom severity, and treatment duration, all recognized as indicators of poor therapeutic response or resistance [3, 4, 47, 48]. Discordant culture results may indicate mixed infections or inadequate empirical antibiotic coverage [4]. Prolonged treatment duration often reflects persistent infection or delayed improvement, while higher severity scores capture more complicated disease courses [45, 46]. Together, these variables reflect known resistance-associated patterns in typhoid. The score was defined as:

$$\begin{gathered} Resistance{\text{ }}Proxy{\text{ }}Score{\text{ }} = {\text{ }} \hfill \\\,\,\,\,\,\,\,\,\,\left( {1{\text{ }}-{\text{ }}Culture{\text{ }}Concordance} \right){\text{ }} \hfill \\\,\,\,\,\,\,\,\,\,\, \times {\text{ }}Normalized{\text{ }}Treatment{\text{ }}Duration{\text{ }} \hfill \\\,\,\,\,\,\,\,\,\,\,\, \times {\text{ }}Severity{\text{ }}Score. \hfill \\ \end{gathered}$$

This assigns higher scores to patients with discordant cultures, longer treatments, and more severe symptoms. Concordant culture cases receive a score of 0, reflecting that matched pathogens across sampled sites at baseline indicate a uniform and microbiologically consistent infection pattern, rather than variability arising from mixed or site-specific pathogens. While not a substitute for antimicrobial susceptibility testing, this score serves as a pragmatic surrogate in data-limited settings [3, 48]. Hemoglobin status was classified as anemic (1), normal (0), or elevated/polycythemia (2) based on sex-specific reference thresholds for biochemical feature engineering; platelet status was coded as thrombocytopenia (1), normal (0), or thrombocytosis (2); calcium status was classified as hypocalcemia (1), normal (0), or hypercalcemia (2); and potassium status was similarly derived as hypokalemia (1), normal (0), or hyperkalemia (2). This allowed raw laboratory values to be converted into clinically interpretable categories for modeling. A derived clinical feature termed “suspected typhoid” was engineered to identify patients who are most likely to have typhoid fever based on a combination of important biochemical indicators, symptom severity, and microbiological evidence. Although definitive diagnosis relies on isolating *Salmonella enterica* serovar Typhi from sterile sites, sensitivity may be reduced due to prior antibiotic use or limited diagnostic capacity in endemic or low-resource settings, resulting in false negatives despite clinical suspicion [45, 49]. To address this, Suspected Typhoid was engineered to include both confirmed and probable cases. It was computed as a binary variable set to (1) for confirmed *Salmonella* presence in blood or urine cultures, or co-occurrence of moderate to high symptom severity (Severity Score ≥ 2) and biochemical signs of infection specifically, anemia (Hemoglobin Status ==1) or thrombocytopenia (Platelet Status ==1), and 0 otherwise. These indicators have been previously associated with typhoid pathophysiology and reflect the systemic impact of the disease [50]. By integrating clinical and laboratory signals, this feature enhances diagnostic sensitivity for identifying probable typhoid cases, especially where culture confirmation is unavailable. The summary is shown in Table S1.

### Metabolomic simulation

Metabolomic simulation, as used in this work, is the computational synthesis of metabolite-related features utilizing clinical and biochemical data to mimic possible metabolic changes in typhoid fever patients. This method supports personalized medicine by approximating host-pathogen metabolic interactions that may impact treatment results or resistance patterns, improves predictive power, and makes multi-omics integration easier by connecting biochemical markers with metabolomics-derived phenotypes. We used a hybrid approach that blends rule-based simulation with machine learning-driven latent profiling to mimic metabolomic layers without the need for direct metabolomic data. Without depending on pre-established pathway linkages or external databases like KEGG, which might not be unique to typhoid fever, our approach provides biological interpretability and catches intricate data patterns. It also lessens the possibility of overfitting that comes with a generative AI model.

#### Rule-based simulation

By mapping variations in biochemical markers to possible metabolic disturbances, the rule-based simulation used regular clinical biochemistry data to infer metabolic disruptions. Six features at the binary metabolite level were simulated: *SimMet_EnergyDisruption*: Marked as 1 if hemoglobin levels were less than 12 g/dL for females or less than 13 g/dL for males, indicating that ATP synthesis was impacted by poor oxygen delivery. If the platelet count was greater than 450,000/μL, *SimMet_InflammatoryStress* was flagged as 1, indicating that systemic inflammation changes metabolic fluxes and cytokine signaling. In *SimMet_MembraneInstability*, calcium values < 8.5 or >10.5 mg/dL were flagged as 1 [51]. If potassium levels were less than 3.5 or greater than 5.0 mmol/L, *SimMet_ElectrolyteDisruption* was flagged as 1, indicating disruptions in cellular metabolism and acid-base balance. If the severity score was ≥ 2, *SimMet_CatabolicBurst* was flagged as 1, indicating elevated protein and energy catabolism linked to systemic inflammation [52]. *SimMet_HypoxiaStress:* Marked as 1 in cases of low hemoglobin levels, this indicates a decreased ability to carry oxygen, which leads to compensatory metabolic adjustments like anaerobic glycolysis [53] and 0 otherwise for all the features. When direct metabolomics profiling is not practical, these features help generate hypotheses, stratify risks, and interpret disease-related states functionally by approximating actual metabolic phenotypes.

#### Unsupervised latent metabolite feature extraction

To extract latent metabolomic features, we applied PCA to a specific set of metabolic data, including clinical biomarkers and indicators of simulated metabolic stress. Before using PCA, all features were standardized using z-score normalization to ensure equal weighting across variables. Two components (n_components = 2) were used in PCA to capture the main axes of variation in the combined clinical and simulated metabolomics profile. The generated principal components (PC1 and PC2) were used as input features in prediction models and functioned as compressed, latent metabolic fingerprints. This strategy is in line with well-established metabolomics techniques, where dimensionality reduction improves prediction accuracy and model generalizability by distilling physiologically significant variance into interpretable components [54–56].

### Model training and optimization

To evaluate predictive performance across clinically relevant outcomes, we developed and optimized three supervised machine learning models using the whole dataset: (i) a binary classifier for treatment outcome, (ii) a regression model for resistance proxy score, and (iii) a classifier for suspected typhoid diagnosis. All models were implemented using the XGBoost algorithm, known for its robustness in structured healthcare data and superior performance in tabular domains [21]. For all models, before training, features with possible leakage and high multicollinearity (based on Pearson correlation analysis) were removed. All Boolean variables were encoded. Hyperparameter tuning was performed using RandomizedSearchCV across 30 sampled configurations, evaluating combinations of learning rate, tree depth, regularization parameters, and sampling ratios, improving model performance and avoiding overfitting [57]. The search space included: n_estimators ∈ {100–500}, max_depth ∈ {3–10}, learning_rate ∈ [0.01–0.3], subsample ∈ [0.5–1.0], colsample_bytree ∈ [0.5–1.0], and reg_lambda ∈ [0–10]. All models were trained using five-fold cross-validation with random shuffling and stratification where applicable. Hyperparameter tuning was executed within the same cross-validation folds used for model evaluation, ensuring that performance metrics reflected only the optimized configuration without introducing external information. The best-scoring configuration from this procedure was refit on the full training data of each seed. For classification tasks, SMOTE was applied to address class imbalance [36] and cross-validation was done using ROC_AUC. SMOTE was applied only to the training partition within each cross-validation split to correct class imbalance in the treatment-outcome and infection-classification tasks. It was implemented using the default k-nearest-neighbor setting (*k* = 5) and the standard oversampling strategy that increases the minority class to match the majority class within each fold. Importantly, SMOTE was *not* applied to the held-out validation splits, thereby preventing information leakage and ensuring unbiased performance estimation. For regression task, the average cross-validated R^2^ was used to choose the final model configuration.

### Model evaluation

For each model, unbiased performance estimates were obtained using a repeated nested cross-validation (nested-CV) framework [58]. To completely separate hyperparameter tuning from performance assessment and prevent optimistic bias during model selection, Nested-CV was selected. 10 separate random seeds (100–109) were used to repeat the whole nested-CV pipeline in order to account for stochastic variation in both data splitting and model initialization. An independent nested-CV procedure with newly generated random splits for the inner and outer cross-validation loops, was run for every seed. Each seed functioned as a separate replication of the whole tuning-and-evaluation process; no models, results, or hyperparameters were exchanged among seeds.

A 5-fold outer cross-validation cycle produced objective evaluations of predicted performance for every seed. A 5-fold inner cross-validation loop was utilized for RandomizedSearchCV hyperparameter adjustment within each outer-fold training split [59]. The hyperparameter search space, which varied in tree depth, learning rate, subsampling ratios, column sampling ratios, number of estimators, gamma regularization, and L2-regularization (λ), was the same for all models. For each inner loop, thirty different random hyperparameter combinations were assessed. The outer loop was strictly reserved for final performance grading, while model selection took place only within the inner loop.

The main optimization measures for XGBoost regressor were mean absolute error (MAE) and coefficient of determination (R^2^) [60]. The area under the receiver operating characteristic curve (ROC AUC) was used to assess model discrimination for XGBoost classifiers. The unbiased assessment of model performance for each seed-level nested-CV run was represented by the mean of the outer-fold scores. Random initialization, random CV partitioning, and stochasticity in the randomized hyperparameter search all contribute to the variability in the final reported performance for each prediction task, which is expressed as mean ± standard deviation among the ten seeds. Repetitive seeds were only used for variance estimates and robustness evaluation; neither ensemble averaging nor model aggregation were carried out [61, 62].

### Drug recommendation simulation

**Fig. 1:**
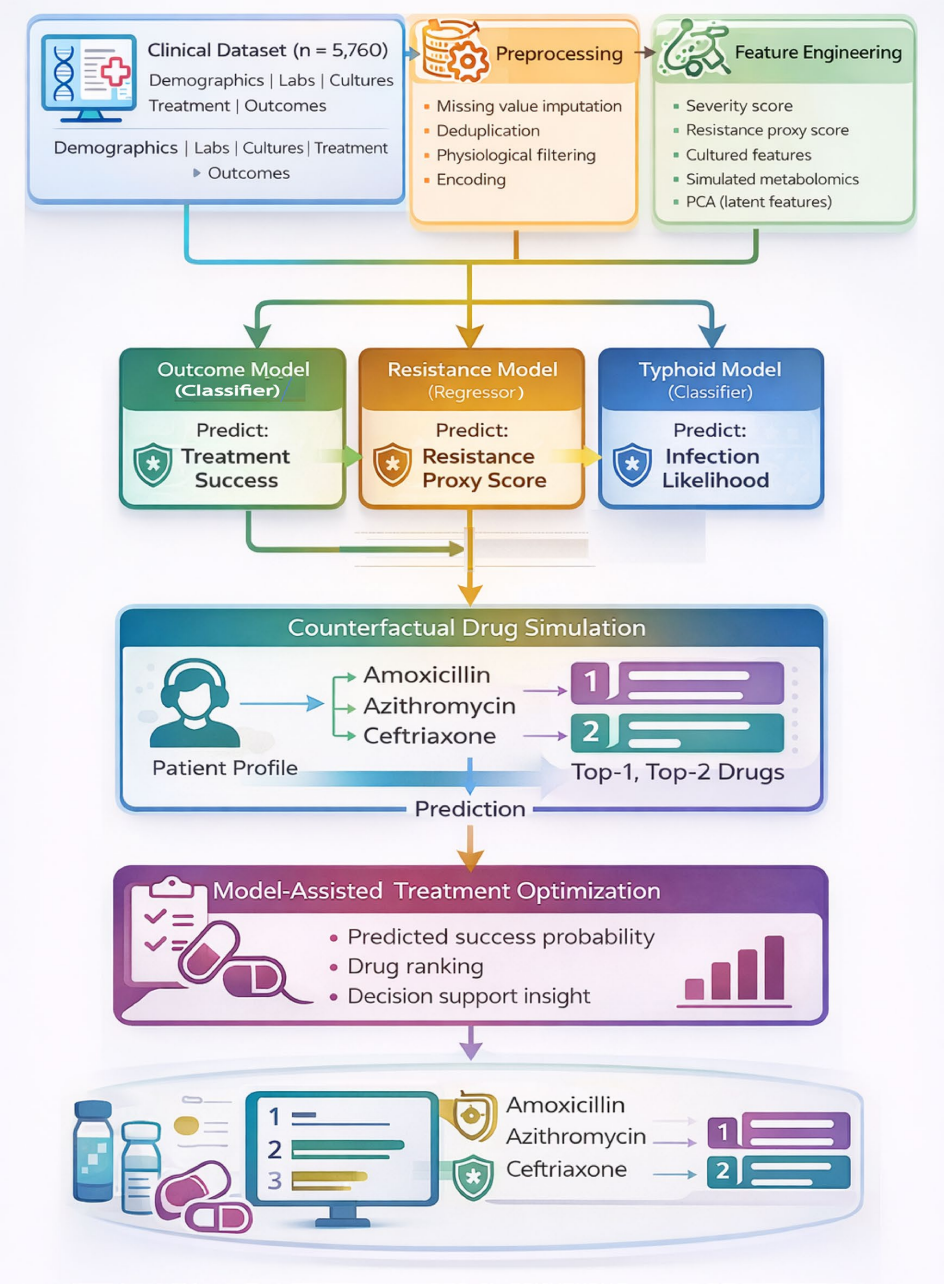
End-to-end study workflow. Clinical and laboratory data were pre-processed, transformed into engineered features, and subsequently used to train multi-task XGBoost models, while a counterfactual drug-simulation module estimated patient-specific alternative antibiotic options

To evaluate the potential for improved treatment outcomes via drug switching, we simulated counterfactual drug assignments for each patient in the test set using the optimized treatment outcome classifier. The simulation aimed to estimate which of three candidate antibiotics, amoxicillin, azithromycin, or ceftriaxone, would maximize the predicted probability of treatment success for each individual, assuming all other clinical variables remained constant. Each patient’s engineered feature vector was replicated three times and manipulated to simulate the administration of each possible antibiotic. Medication features were encoded in a mutually exclusive fashion to reflect realistic prescribing practice. All features were passed through the outcome classifier, which returned predicted probabilities of treatment success. The top two ranked antibiotics (by predicted success probability) were retained for analysis. The resultant enriched dataset allowed for retrospective comparison between real-world prescribing and model-recommended alternatives. The resultant predictions were subjected to further downstream comparative analysis. This study was conducted and reported in accordance with the TRIPOD-AI reporting recommendations, where applicable. The whole method flow is shown in Fig. 1.

## Results and discussion

### Dataset overview

The final analytical cohort consisted of 1,659 patient records following preprocessing and harmonization (Section “Data acquisition and pre-processing”). The population exhibited a near-balanced sex distribution, with males accounting for 51.3% of patients. The mean age was 42.6 years (SD = 15.04; range: 18–70 years), consistent with the adult demographic typically affected in endemic settings. Laboratory parameters were broadly within physiological ranges, with a mean hemoglobin concentration of 12.49 g/dL (SD = 1.46), suggesting the presence of mild or subclinical anemia in a subset of patients. The mean platelet count was 209,115/µL (SD ≈ 49,861), while mean calcium and potassium concentrations were 9.02 mg/dL and 4.29 mmol/L, respectively. The average treatment duration was 9.97 days (SD = 2.71), aligning with standard therapeutic regimens for uncomplicated typhoid fever (Supplementary File 2, Sheet: Statistics).

Categorical variables demonstrated clinically coherent distributions. Symptom severity spanned four categories, with low severity representing the largest group (*n* = 478). Blood culture results were dominated by “Unknown” profiles (*n* = 491), followed by isolates including *Salmonella typhi*, *Staphylococcus* spp., and *Escherichia coli*. The predominance of *S. typhi* is consistent with its etiological role in enteric fever, while the presence of other organisms likely reflects co-infections or diagnostic overlap in febrile illness presentations [3, 4]. Urine cultures were primarily characterized by *E. coli* (*n* = 586), suggesting a substantial burden of concurrent urinary tract infections. Antibiotic prescription patterns reflected contemporary clinical practice. Amoxicillin (*n* = 610) was the most frequently administered agent, followed by ceftriaxone and azithromycin (Fig S1). This distribution underscores the continued reliance on legacy antibiotics alongside newer agents in the context of evolving antimicrobial resistance, highlighting the clinical relevance of data-driven treatment optimization strategies [47].

Correlation analysis of continuous variables revealed no evidence of multicollinearity, with all pairwise Pearson coefficients remaining negligible (|r| <0.06). This indicates minimal redundancy among predictors and supports the use of non-linear machine learning approaches capable of leveraging independent feature contributions. The absence of strong linear dependencies further suggests that predictive performance is driven by multivariate interactions rather than simple pairwise associations (Fig. S2).

### Feature engineering

Robust modeling of typhoid-related clinical variability was made possible by the conversion of raw clinical, biochemical, and microbiological information into analytically tractable and physiologically meaningful engineered features. These engineered features consistently displayed patterns that matched their intended semantic definitions, as seen in Fig. S3. There was no discernible imbalance or propagation of missing data, and key categorical features maintained logical distributions [49]. Clinical variability among the patient population was shown by the multimodal or right-skewed distributions of continuous designed variables [45, 63, 64].

#### Simulated metabolomic profile distributions

The six generated features showed distinct distribution patterns, as seen in Fig. S3. SimMet_EnergyDisruption and SimMet_HypoxiaStress were common, indicating anemia that is frequently seen in systemic infections, such as typhoid [65]. In line with cytokine-driven platelet increases documented in typhoid fever [66], SimMet_InflammatoryStress identified a subpopulation with increased inflammatory activity. SimMet_ElectrolyteDisruption and SimMet_MembraneInstability were less prevalent [4]. In the absence of direct metabolomics testing, these characteristics provided significant approximations of underlying metabolic abnormalities and were both clinically interpretable and in line with recognized biochemical thresholds.

#### Dimensionality reduction and latent biochemical signatures

**Fig. 2:**
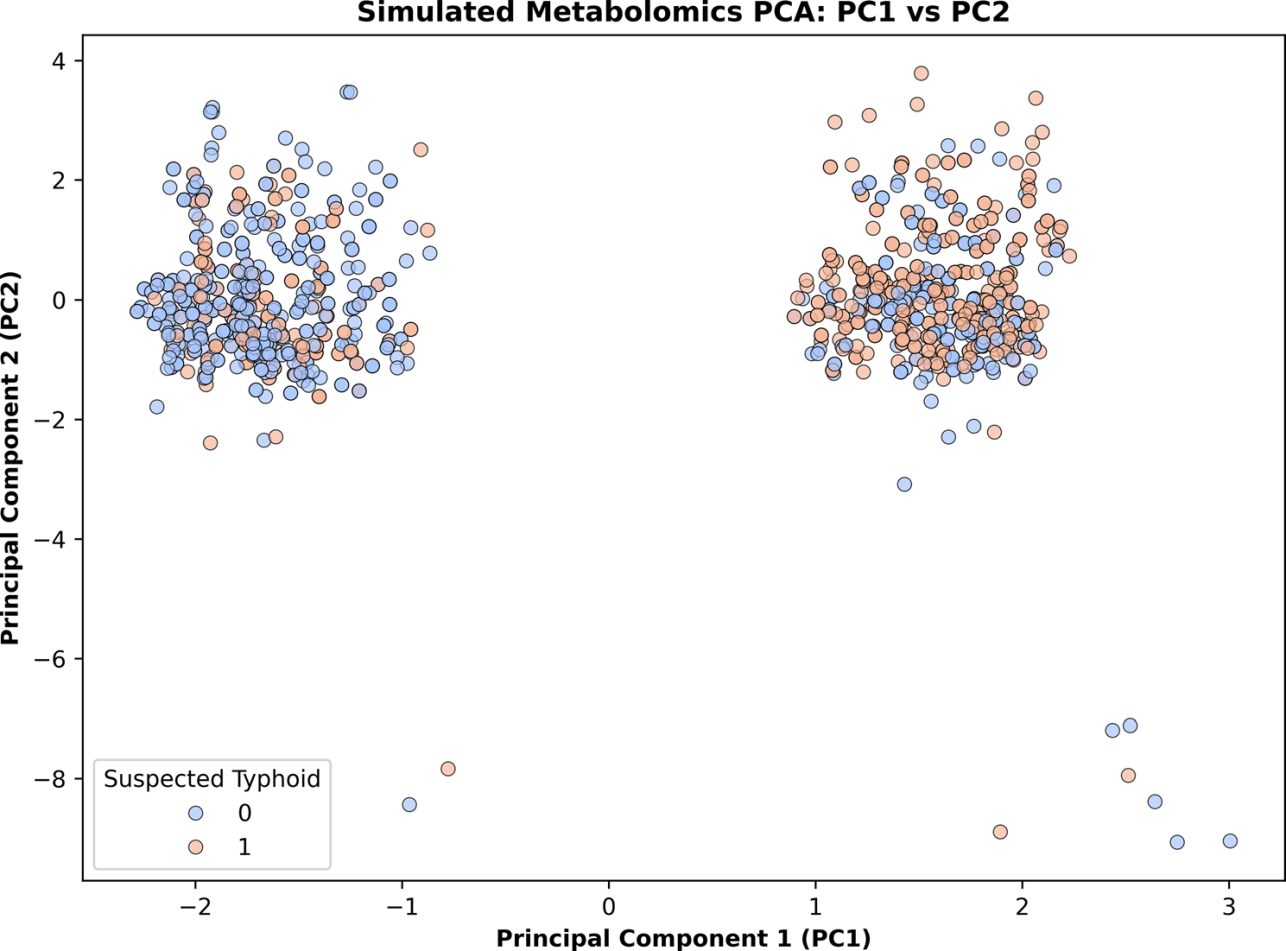
PCA projection of metabolomic and biochemical feature space

Using PCA on both raw biochemical markers and simulated metabolomics features, two notable latent components (PC1 and PC2) were identified (Fig. 2). A systemic illness load was indicated by PC1‘s weighting primarily on anemia and inflammatory signs, but PC2‘s representation of fluctuations in membrane and electrolyte stability suggested renal or mitochondrial involvement. Orthogonal tendencies were noted between the constituent parts. The dimensionality reduction preserved significant biological structure, as seen by the grouping of suspected or confirmed typhoid patients along particular PC1–PC2 space regions. This approach mirrors conventional metabolomics workflows, where PCA is used to compress high-dimensional data while maintaining key biological variance [54, 67]. More precise modeling was made possible by this extensive feature set.

### Model overview and training performance

The final set of prediction models was developed using a 75:25 training set to test set ratio using clinical datasets that had been carefully engineered, deduplicated, and cleaned. A customized feature set was used as input for each model. The machine learning framework used stratified 5-fold cross-validation over 30 hyperparameter configurations per model and grid search to achieve 150 fits per task. In clinical prediction tasks, XGBoost was used as the basis estimator because of its effectiveness and scalability [21].

#### Treatment outcome model

The model was trained on data from 963 patients to predict binary treatment outcomes after 281 rows with missing values were removed during preprocessing. The original dataset showed a moderate class imbalance (success: failure ~1.19:1) and created a balanced training set during model training. Laboratory results and metabolomic simulations were among the 21 features that were used in the final model. With a cross-validated ROC AUC of 0.9479, the model demonstrated strong discrimination. The best configuration used n_estimators = 300, max_depth = 6, learning_rate = 0.05, subsample = 0.8, reg_lambda = 0.1, gamma = 0, and colsample_bytree = 1.0 [68].

#### Resistance proxy model

The final model was trained on 963 clean observations after excluding 281 rows with missing score components. The feature set (*n* = 20) included antimicrobial exposure, laboratory metrics, and simulated metabolomic features. The model achieved a cross-validated R^2^ of 0.5979, confirming a moderate approximation of resistance likelihood based on engineered proxies. The best parameters were n_estimators = 300, max_depth = 8, learning_rate = 0.1, subsample = 0.9, colsample_bytree = 1.0, gamma = 3, and reg_lambda = 1 [45, 63].

#### Suspected typhoid model

To identify instances of clinically suspected typhoid, an approximately balanced dataset (*n* = 963) was used with 20 features spanning clinical, biochemical, and simulated metabolomic attributes. The model demostrated strong discrimination with a cross-validated ROC AUC of 0.9182. The final hyperparameters were n_estimators = 300, max_depth = 10, learning_rate = 0.1, subsample = 0.9, colsample_bytree = 0.7, gamma = 0, and reg_lambda = 10 [65].

### Test set evaluation and generalization

The performance of all models was assessed across ten separate runs using a bootstrap-based resampling technique on a held-out test set of 415. This evaluation strategy enhances robustness and quantifies generalization error by incorporating variability introduced by random sampling [69].

#### Treatment outcome classification

**Fig. 3:**
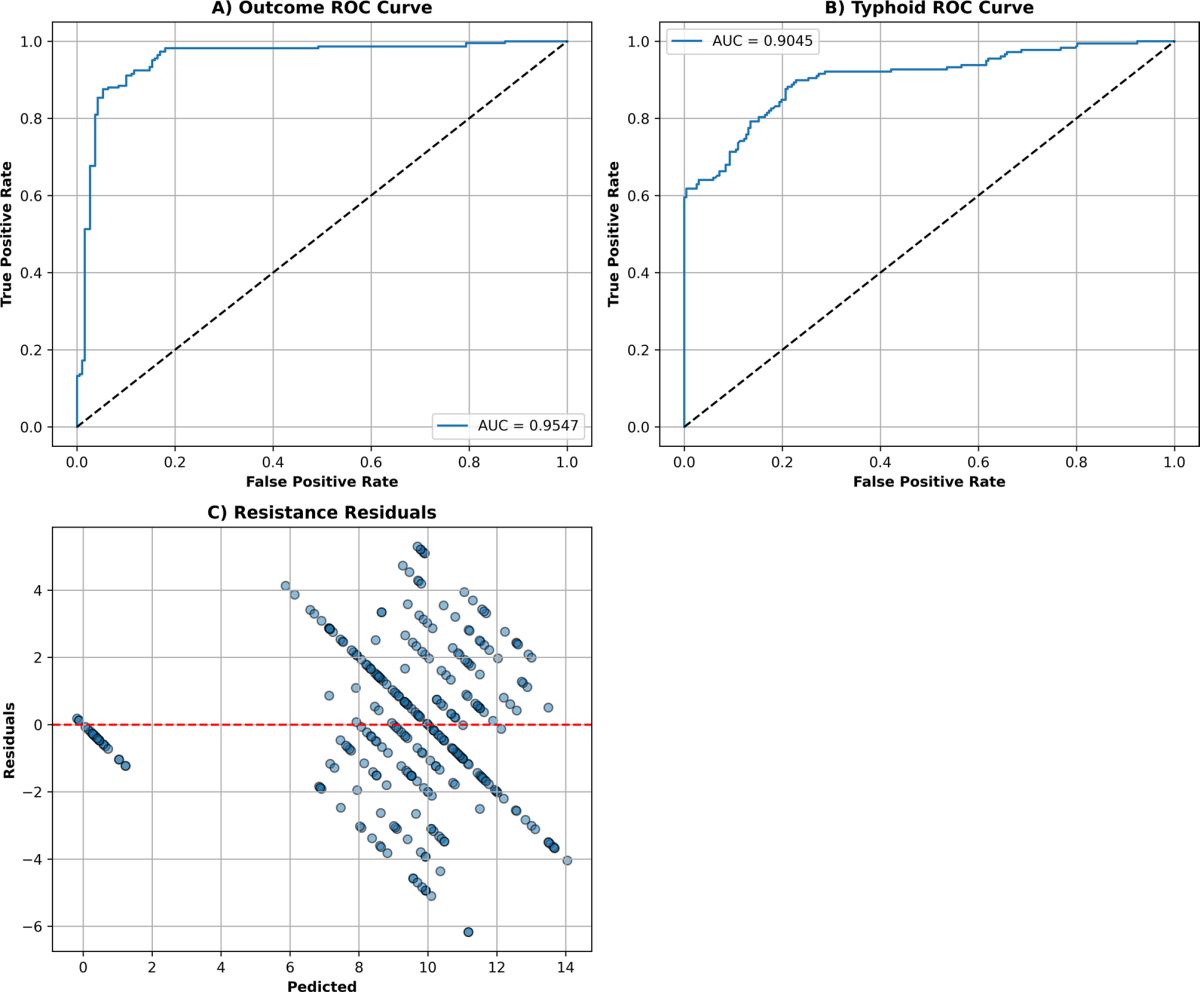
ROC and residual plots for classification and regression models. (**A**) and (**B**) show ROC curves for the outcome and suspected typhoid classifiers, respectively, indicating strong discriminative ability. (**C**) displays residual plot for resistance proxy regression model, showing tight error distributions without systematic deviation

With an average ROC AUC of 0.9624 ± 0.0099, the outcome classifier demonstrated strong generalization performance (Table 1 and Fig. 3A). On the test set (*n* = 415), the total classification accuracy was 90%, and both positive and negative classifications performed consistently (precision ≈ 0.89–0.91; recall ≈0.89–0.91). Strong stability is reflected by the macro-averaged F1-score of approximately 0.90 and the relatively low variance across bootstrap folds (Table 2). These results bolster the clinical outcome prediction’s external validity and robustness. Reliable class identification and a clear predictive structure in the feature space are suggested by high ROC AUC values (Fig. 3A). The confusion matrix (Fig. S4A) demonstrates strong concordance between observed and predicted labels, with true negative (*n* = 169) and true positive (*n* = 206) predictions substantially outnumbering incorrect predictions (false negatives = 20, false positives = 20).

**Table 1:** Evaluation metrics for all predictive models on the held-out test set using repeated cross-validation and bootstrap resampling. All models demonstrate stable generalization performance

Task	Metric	Mean ± SD
Resistance Proxy (regression)	R^2^	0.5878 ± 0.0112
	MAE	1.8451 ± 0.0228
	MSE	5.1829
	RMSE	2.2766
Treatment Outcome (classification)	ROC AUC	0.9624 ± 0.0099
Suspected Typhoid (classification)	ROC AUC	0.9018 ± 0.0045

**Table 2:** Precision, recall, F1-score, and accuracy for binary classification tasks on the test set. The outcome model performs slightly better overall, while the typhoid model maintains balanced class-wise metrics

Model	Class	Precision	Recall	F1-score	Support
Outcome	0.0 (Failure)	0.89	0.89	0.89	189
	1.0 (Success)	0.91	0.91	0.91	226
	Accuracy			0.90	415
	Macro Avg	0.90	0.90	0.90	
	Weighted Avg	0.90	0.90	0.90	
Typhoid	0 (Negative)	0.81	0.89	0.85	237
	1 (Positive)	0.83	0.72	0.77	178
	Accuracy			0.82	415
	Macro Avg	0.82	0.81	0.81	
	Weighted Avg	0.82	0.82	0.82	

#### Resistance proxy regression

The resistance proxy model also demonstrated consistent generalization performance, with an average R^2^ of 0.5878 ± 0.0112 and MAE of approximately 1.8451 ± 0.0228. Despite the engineered nature of the target, evaluation on unseen data yielded moderate predictive accuracy, as supported by regression diagnostics (MSE: 5.1829, RMSE: 2.2766). Although proxy variables are not substitutes for lab-confirmed antimicrobial resistance, their predictive performance reinforces their value in low-resource simulation, risk stratification, and early warning systems [70]. Resistance residuals (Fig. 3C) displayed wider dispersion and clustered residual deviations, particularly at higher predicted scores. Such patterns suggest that while the resistance model captures broad outcome tendencies, additional explanatory variables such as pathogen-specific genomic features or prior antimicrobial exposure history may be required to reduce unexplained variance.

#### Suspected typhoid classification

The overall accuracy of the suspected typhoid classifier was 82%, with a robust average ROC AUC of 0.9018 ± 0.0045 (Table 1 and Fig. 3B). Positive and negative class-specific F1-scores were 0.77 and 0.85, respectively, and macro-averaged precision and recall remained above 0.81 (Table 2). Such a model offers critical utility in endemic areas [4, 63]. The legitimacy of the constructed pipeline and the viability of proxy-enhanced modeling in infectious disease contexts were supported by the stable performance of all models across numerous bootstrap runs. The confusion matrix (Fig. S4B) shows that the typhoid classification model achieved good overall accuracy, correctly identifying 211 negative cases and 129 positives, with false positives (*n* = 26) and false negatives (*n* = 49). The evaluation indicates that both outcome and typhoid classifiers achieve reliable categorical performance with moderate misclassification, reflecting the inherent clinical variability in infectious disease diagnosis.

It is important to note that the dataset originally comprised only patients who were clinically managed as typhoid-positive cases. Consequently, the negative class used in model training and evaluation was derived through feature engineering rather than direct clinical annotation. Specifically, patients who did not satisfy the composite “suspected typhoid” criteria were designated as negatives, despite being considered positive by clinicians in routine practice. This distinction underscores that the classifier’s performance reflects separation between “probable typhoid” and “less consistent typhoid presentations,” rather than true positives versus true negatives in the strict diagnostic sense. While this limits direct comparability with gold-standard microbiological definitions, it also highlights an important contribution: the model identifies internal structure within a uniformly positive dataset, effectively distinguishing subsets of patients who may be over-classified as typhoid in frontline practice. Moreover, by producing probabilities rather than absolute labels, the classifier provides a spectrum of likelihood, offering a more nuanced view of case heterogeneity than a binary clinical approach. In this way, the approach demonstrates how computational methods can reveal hidden diagnostic heterogeneity and potentially refine clinical case definitions in endemic settings.

### Model calibration and probability reliability assessment

When making decisions at the individual or community level, calibration establishes how reliable the anticipated probabilities are [71]. To evaluate the dependability of probabilistic results from predictive models, we used scikit-learn’s calibration curve analysis [72] to perform a post hoc calibration aassessment. For each model, predicted class probabilities were generated and calibration curves were constructed using 10 equal-width bins. These curves plot the mean predicted probability against the observed fraction of positive outcomes. A reference line (y = x) was included to indicate perfect calibration, where predicted probabilities align exactly with observed empirical frequencies. Deviations from this diagonal reflect over- or under-confidence in the model outputs. In addition, the Brier score, a proper scoring rule for probabilistic predictions, was computed to quantify overall calibration quality [73].

**Fig. 4:**
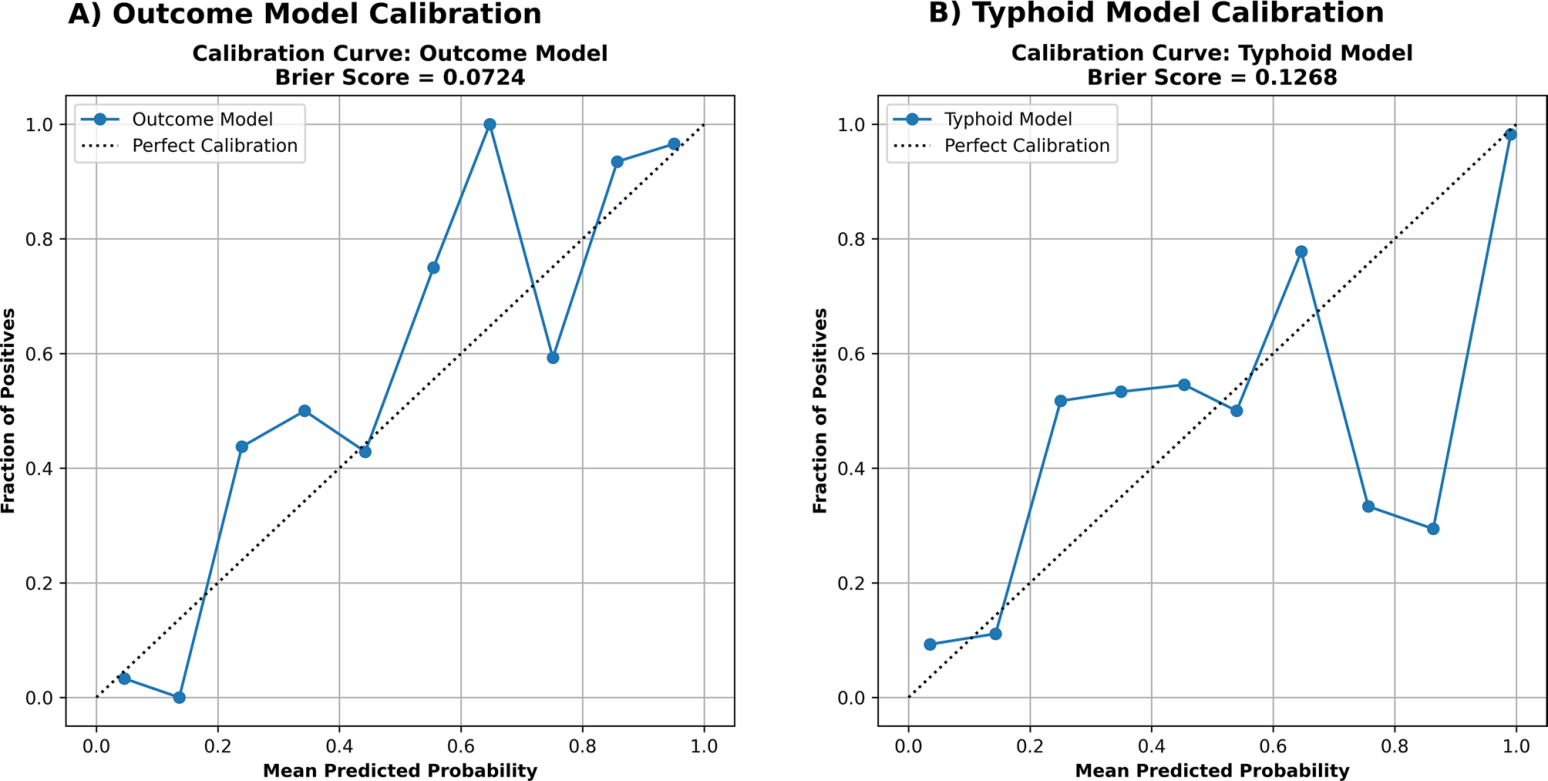
Calibration plots of predictive classification models. The treatment outcome model (**A**) demonstrated good overall calibration, while the suspected typhoid model (**B**) showed moderate calibration with increasing deviation at higher predicted probabilities. The dashed diagonal in both panels represents perfect calibration

The Outcome model’s low Brier score (0.0724) indicates relatively small discrepancies between predicted and observed outcomes, consistent with acceptable calibration performance in clinical prediction models [74]. This calibration level suggests reasonable uncertainty quantification alongside good discriminative perfomance (Fig. 4A). With a Brier score of 0.1268, the Typhoid model (Fig. 4B) demonstrates lower calibration performance than the outcome model but still remains within a range considered usable for many public health applications, albeit with greater prediction error [72].

Model calibration for continuous predictions was further assessed via Q-Q plots (Fig. S4C). For resistance scores, the distribution shows noticeable departures from the theoretical normal quantiles, particularly toward the upper tail, where values diverge more strongly from the expected linear trend. This curvature indicates deviations from normality in the residual distribution and may reflect underlying heterogeneity in resistance determinants or nonlinear relationships not fully captured by the model. Despite these deviations, the overall pattern still provides useful insight into model behavior and suggests that incorporating additional biological or clinical covariates could further improve resistance prediction performance.

### Model explainability and SHAP-based patient stratification

To enhance the interpretability of the trained machine-learning models, SHAP analysis was performed [75]. For each task, the corresponding XGBoost model [21] and its specific test feature set were loaded.

Features were aligned and padded where necessary to ensure compatibility with the trained model. SHAP values were computed using TreeExplainer, which is optimized for gradient-boosted tree models. The top 20 features were extracted based on mean absolute SHAP values and visualized using beeswarm plots to summarize both global and feature-level contributions [75]. To examine feature consistency across predictive tasks, overlaps in top features were quantified and visualized using a Venn diagram and pairwise heatmap [76]. To assess patient-level similarity in model explanations, SHAP value matrices across the three tasks were concatenated per patient. The combined matrix was standardized using z-score normalization and subsequently clustered using KMeans (k = 5). Dimensionality reduction was performed via Uniform Manifold Approximation and Projection (UMAP) [37] and t-distributed Stochastic Neighbor Embedding (t-SNE) to visualize latent patient subgroups based on SHAP attribution signatures.

#### Global feature importance across models

**Fig. 5:**
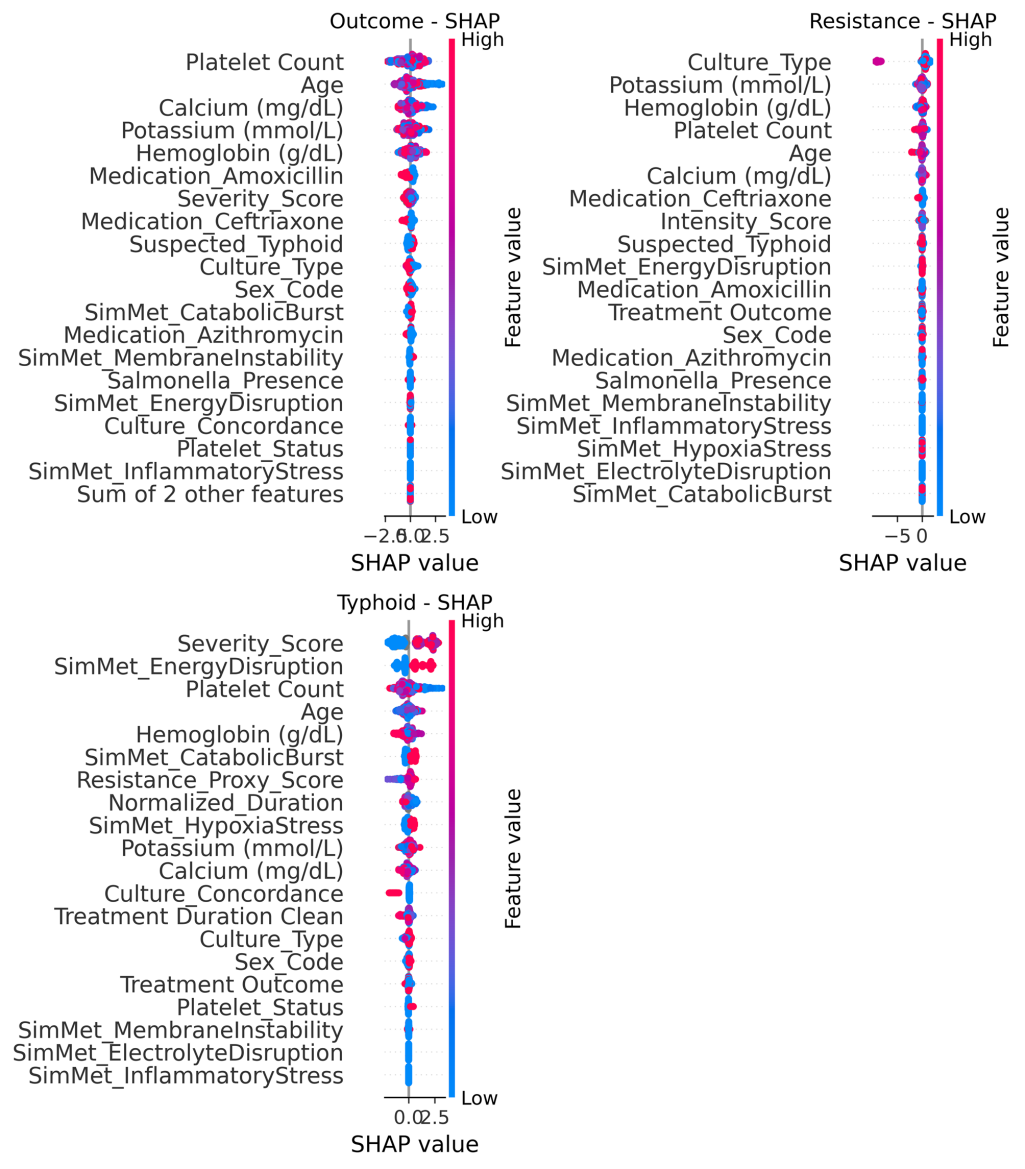
SHAP beeswarm plots for the three predictive models. Each plot displays the top 20 most influential features based on their SHAP values, which reflect their impact on the model’s predictions. Color gradients represent the original feature values

SHAP analysis showed that each of the three prediction models exhibited model-specific yet biologically relevant feature importance patterns (Fig. 5, Table S2). The treatment outcome model’s primary predictors were platelet count, age, calcium (mg/dL), potassium (mmol/L), and hemoglobin (g/dL), highlighting the significance of hematologic and biochemical status in assessing treatment efficacy. Notably, Severity_Score and Culture_Type also contributed meaningfully, supporting the view that clinical severity and pathogen characteristics influence therapy response [4, 76]. Platelet count and culture_type were among the main drivers of the resistance proxy regression model, followed by potassium (mmol/L), hemoglobin (g/dL), age, and calcium (mg/dL). Several simulated metabolic stress traits, including SimMet_EnergyDisruption and SimMet_MembraneInstability, also appeared among influential features, highlighting the potential role of microbial physiological stress states in shaping resistance patterns. These findings are broadly consistent with established associations between antibiotic resistance, microbiological profile variation, and culture discordance. Lastly, Severity_Score was the strongest driver in the suspected typhoid classification model, followed by SimMet_EnergyDisruption, Platelet Count, Age, and Hemoglobin (g/dL). The presence of Resistance_Proxy_Score further suggests that systemic stress, inflammation, and hematologic disruption contribute to the diagnostic signal captured by the model, consistent with clinical observations of cytopenias and metabolic stress in enteric fever.

#### Cross-model feature overlap and modularity

The Venn diagram demonstrates substantial overlap in the top-ranked predictors among the Outcome, Resistance, and Typhoid models, with 12 features shared across all three models. Pairwise overlaps were also prominent, with five features shared exclusively between the Outcome and Resistance models, three between Outcome and Typhoid, and two between Resistance and Typhoid. Only minimal model-specific signals were observed, with one feature unique to the Resistance model and three unique to the Typhoid model, while the Outcome model showed no exclusive predictors among its top features.

**Fig. 6:**
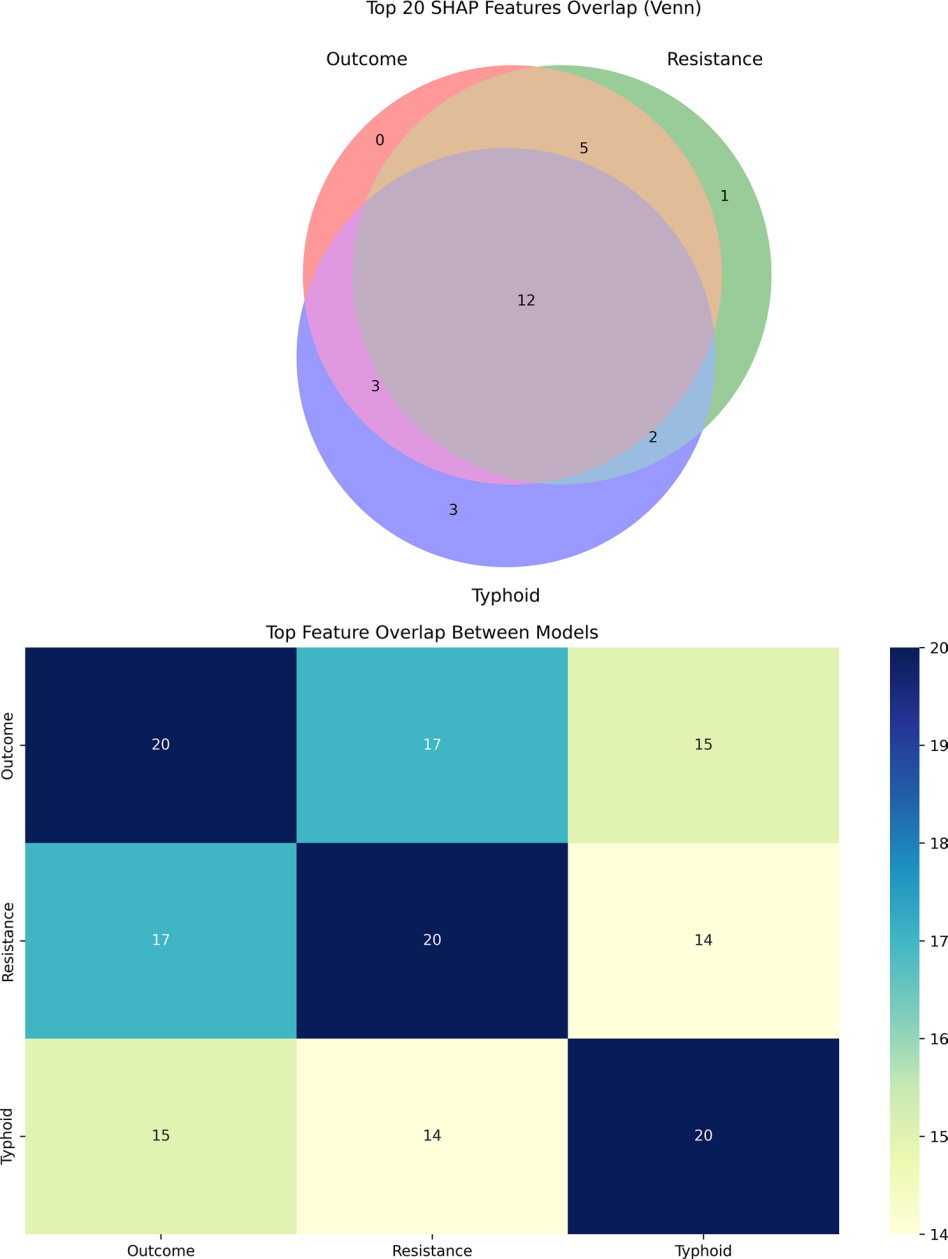
Cross-model SHAP feature overlap across predictive tasks. Venn diagram (top) and pairwise overlap heatmap (bottom) showing the number of shared top-ranked SHAP features across the three predictive models. Common predictors such as platelet count, age, PC1, and hemoglobin (g/dL) are recurrent across models, while a small subset remains task-specific. This pattern reflects a balance between shared physiological drivers and modular task-specific signals

Despite this convergence, each model retained some degree of task-specific predictive signal. Simulated metabolic indicators such as SimMet_EnergyDisruption and SimMet_CatabolicBurst remained particularly influential in Typhoid prediction, reflecting more specialized metabolic stress markers likely associated with infection severity. The pairwise overlap heatmap further quantifies this pattern. The Outcome and Resistance models shared 17 of their top features, while Outcome and Typhoid shared 15, and Resistance and Typhoid shared 14, indicating strong cross-model physiological consistency while maintaining modest task-specific divergence. This pattern reflects the modular yet interdependent nature of the models, where shared predictors enable synergistic learning across tasks while preserving model-specific inputs that allow task-specific optimization. Such a structure is particularly valuable in clinical settings were overlapping but non-identical decision goals must be simultaneously addressed (Fig. 6).

#### Patient-level explanations and personalized attribution

To complement global feature-importance analyses, patient-level explanations were generated using SHAP for all three predictive models. Each model was visualized through standardized layouts combining summary plots and individual waterfall plots (Figures S5–S7), which together illustrate how global feature contributions translate to personalized predictions. This methodology follows best practices for explainable model interpretation and supports individualized insight into complex ensemble decisions [39]. In the Treatment Outcome model (Fig. S5), the individualized attribution plot indicates that platelet count, potassium, and age positively contribute to the model’s results, collectively shifting the probability toward recovery and reinforcing their relevance as interpretable clinical markers of prognosis.

The Resistance model (Fig. S6) showed a greater reliance on culture type in the negative direction, alongside biochemical features, particularly calcium and potassium, and hematological features such as platelet count in the positive direction. These markers influenced the individualized resistance score in a manner consistent with reported associations between culture type, electrolyte imbalance, and antimicrobial resistance phenotypes [28, 31]. Finally, for the Typhoid classification model (Fig. S7), the SHAP patient-level explanation shows that SimMet_EnergyDisruption contributed positively to the model, whereas the severity score showed a negative contribution to the prediction. Normalized duration and potassium also contributed negatively in the patient-level waterfall plot, providing a transparent account of how the model aggregated clinical and demographic signals into a binary decision.

The SHAP-based analysis confirms that interpretable modeling can be achieved in multi-task infectious disease prediction without compromising predictive power. Model-specific emphasis, such as the Resistance model’s focus on culture type versus the Typhoid classifier’s integration of broader severity-related variables, suggests that different predictive tasks leverage distinct, but clinically plausible, biological pathways. The strong influence of hematological (Platelet Count, Hemoglobin) and biochemical features (Calcium, Potassium) reflects their central role in patient stratification and treatment response estimation, corroborating findings from prior clinical studies on typhoid and sepsis outcomes while extending interpretability frameworks into the domain of personalized infectious disease prediction [77]. The consistent prominence of platelet count across all models underscores its potential as a unifying biomarker for infection severity, treatment response, and recovery trajectory.

#### Attribution-based patient clustering

**Fig. 7:**
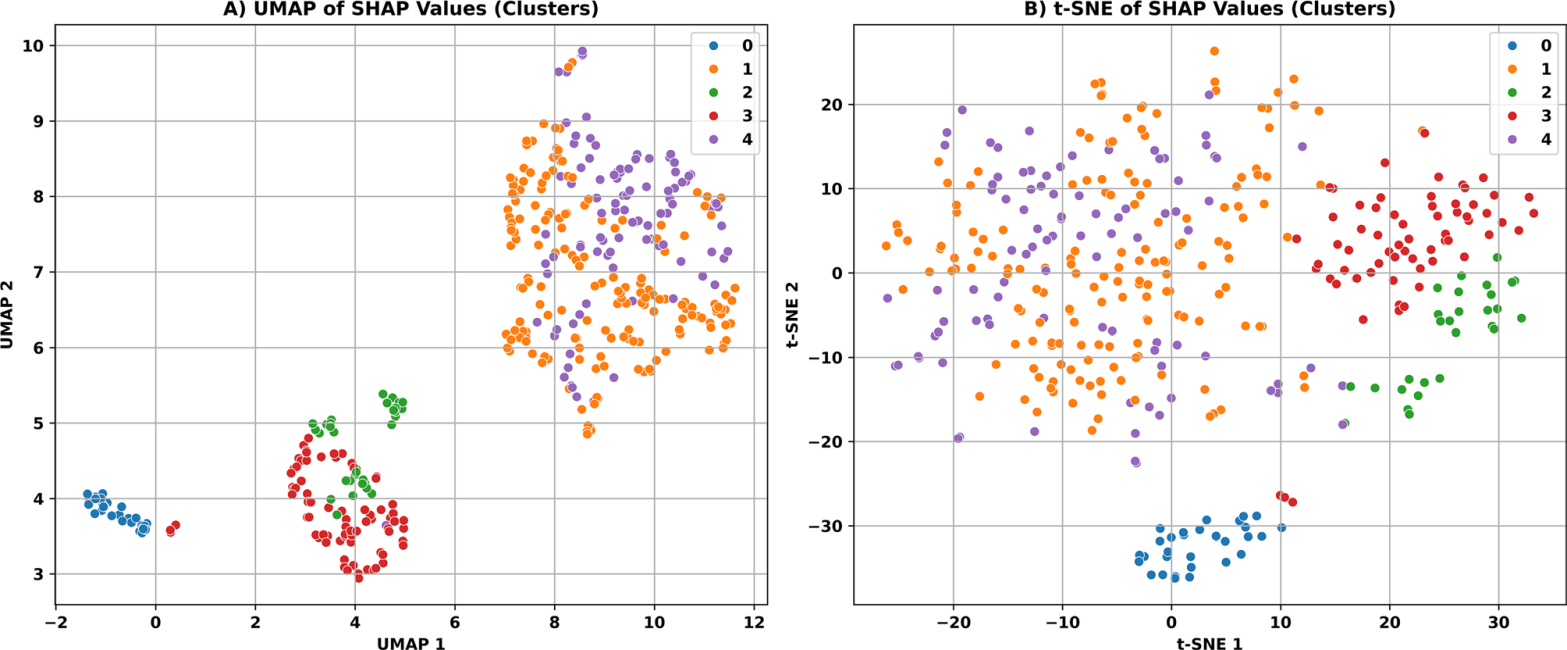
Low-dimensional projection of patient-level SHAP value vectors across all three predictive models using UMAP (left) and t-SNE (right). Each point represents a patient colored by cluster membership derived from attribution patterns. Distinct cluster separation indicates the presence of latent patient subgroups characterized by similar combinations of explanatory feature contributions across the models

By concatenating SHAP value vectors across all three models, we projected patient-level explanatory profiles into two dimensions using UMAP and t-SNE (Fig. 7). Both embeddings revealed distinct, reproducible clusters representing latent patient subgroups defined by shared prediction logic rather than raw clinical inputs. In the UMAP projection, several compact and well-separated clusters are visible, indicating that patients with similar attribution patterns group together in the reduced feature space. The t-SNE embedding similarly preserves this structure while spreading clusters across the projection plane, further supporting the stability of the learned attribution patterns. Across both methods, five primary clusters were observed, suggesting the presence of latent patient subgroups characterized by common combinations of clinical, biochemical, and model-derived signals. For example, some clusters appear tightly grouped and well separated from the others, indicating patients whose predictions are driven by similar explanatory feature profiles, while other clusters show partial dispersion, reflecting greater heterogeneity in the underlying feature contributions. The consistency of clustering patterns across both embedding techniques supports the robustness of these attribution-derived patient groupings. This analysis demonstrates how explainability-informed clustering can uncover clinically meaningful substructures within heterogeneous infectious disease cohorts. Such an approach extends beyond model performance to enable AI-assisted triage, hypothesis generation, and the identification of explainability-guided patient subtypes, in line with emerging literature on interpretable machine learning for precision medicine [78].

The integration of SHAP-based explainability in the present framework aligns with the growing application of explainable artificial intelligence in infectious disease modeling. Recent studies have demonstrated the utility of XAI methods in supporting clinical interpretation of machine learning models for febrile illness diagnosis and infectious disease risk stratification [19, 79, 80]. These approaches have highlighted the importance of interpretable models in clinical environments, particularly where trust, transparency, and accountability are critical for adoption. However, most existing XAI applications in infectious diseases have primarily focused on diagnostic classification or disease detection tasks. In contrast, the present study extends the application of explainable machine learning to treatment decision support, incorporating interpretable models for predicting treatment outcomes and resistance risk while simultaneously evaluating alternative antibiotic strategies through counterfactual drug simulations. By combining multi-task predictive modeling with SHAP-based interpretability, this framework provides both predictive insight and actionable clinical guidance, thereby expanding the role of XAI from diagnostic support toward therapeutic optimization in infectious disease management.

### Drug simulation recommendations

#### Model-wide and per-drug SHAP interpretability

**Fig. 8:**
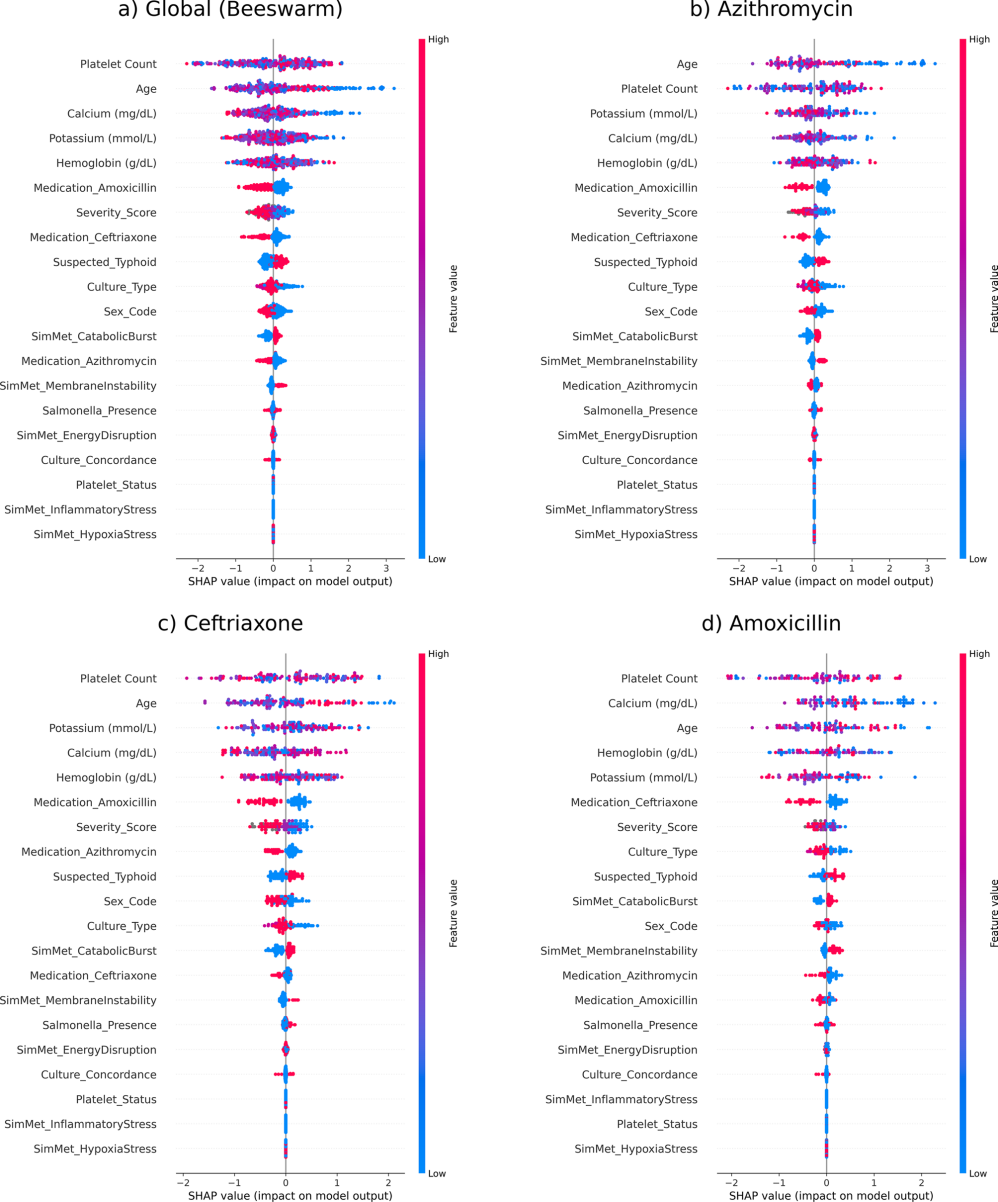
SHAP beeswarm plots showing feature contributions to treatment outcome predictions globally (**a**) and stratified by top-1 predicted drug: azithromycin (**b**), ceftriaxone (**c**), and Amoxicillin (**d**)

Using the same underlying outcome model, we grouped patients by their recommended drug and used SHAP analysis to evaluate the model’s descision logic within each subgroup. Figs 8 and S8 show the results for both global and drug-specific explanations of prediction behavior after simulating treatment outcome probabilities for each patient across all candidate antibiotics. Consistent with the updated model results, the dominant predictors across the analysis remained platelet count and age, followed by key physiological laboratory markers including calcium, potassium, and hemoglobin, though their relative importance varied slightly across drug-specific subgroups. These findings indicate that the model’s recommendations are largely driven by patient physiological state rather than treatment assignment alone.

This analysis reveals both shared and drug-specific patterns in treatment decision logic. Consistently across all groups, Platelet Count and Age were the most influential features, though their relative ranking differed slightly by treatment scenario. For Azithromycin, Age showed the strongest contribution, followed by Platelet Count and electrolyte indicators such as potassium and calcium. In contrast, Ceftriaxone and Amoxicillin predictions were primarily driven by Platelet Count, with Age and electrolyte-related variables contributing secondary influence.

Stratified analysis revealed additional differences in feature weighting across drugs. Electrolyte markers such as calcium and potassium appeared more prominent in Amoxicillin and Ceftriaxone scenarios, while Age contributed more strongly to predictions under Azithromycin recommendations. Across all groups, medication indicators and severity score contributed moderate explanatory value, reflecting interactions between baseline physiology and treatment assignment. In contrast, culture outcomes and simulated metabolomic stress indicators consistently showed low SHAP contributions, suggesting that under the current modeling configuration these variables add limited predictive information relative to core physiological markers. This may reflect redundancy with major clinical variables or limited signal in the available dataset.

#### Top vs actual drug

We compared the top predicted medications with observed treatment records to assess the degree of agreement between model-generated drug recommendations and real clinical prescriptions. The evaluation contrasted the actual drug /clinicians prescribed drug with the Top Drug prediction (Top1 Match means the Top1_Drug matches the Actual_Drug, Top2 Match means the Top2_Drug matches the Actual_Drug, but Top1_Drug does not, and No Match means neither the Top1_Drug nor the Top2_Drug matches the Actual_Drug), which was the model’s main recommendation. Using a weighted average technique to account for class imbalance, we calculated important classification measures such as accuracy, precision, recall, and F1-score. Additionally, Seaborn’s heatmap tool was used to show the confusion matrices. This analysis captures not only exact Top-1 matches but also near-miss recommendations where the correct drug appeared as the model’s second-best suggestion, which is valuable in multi-option clinical decision-making contexts.

The final distribution of match types was summarized using absolute counts and normalized percentages to facilitate comparison across categories. To further evaluate how alignment between model-predicted drug recommendations and clinically administered drugs relates to treatment outcomes and key numeric indicators, and why the model may recommend a different drug where the actual outcome was successful, and find out whether it actually prioritized success probability, lower treatment duration, or resistance proxy score, we performed a stratified analysis based on match type. Subsequent analysis focused on comparing treatment success rates and quantitative prediction metrics across match types. Descriptive statistics (mean, standard deviation, and count) for these variables were computed per match type. A success rate was calculated as the proportion of patients in each group for whom the Treatment Outcome was labelled as successful. This analysis was designed to uncover systematic differences in predicted and real-world outcomes across model confidence levels, providing insight into the practical reliability of the Top-K drug recommender system. This type of comparative outcome analysis is commonly used in clinical decision support validation studies to assess model utility under partial match scenarios and helps contextualize predictive performance beyond standard classification metrics [81, 82].

In 37.11% of cases, the model’s Top-1 predictions matched the actual medication that was provided, whereas in 28.19% of cases the true medication appeared as the model’s second-best recommendation. In 34.70% of cases, neither of the model’s top two predictions matched the prescribed medication. The Top-1 classification achieved an overall accuracy of 0.3711 with a weighted F1-score of 0.3682. At the class level, Azithromycin showed the strongest recall (0.49), indicating that the model most frequently identified this drug correctly when it was prescribed. Ceftriaxone demonstrated moderate performance (F1-score 0.33), while Amoxicillin showed lower recall (0.28) despite moderate precision (0.47). When evaluating the second-ranked recommendation, the Top-2 classification showed reduced performance, with an overall accuracy of 0.2819 and an F1-score of 0.2863. Across drugs, performance remained modest, suggesting that incorporating the second-ranked prediction alone does not substantially improve classification agreement with historical prescriptions (Tables S3–S4; Figs. S9–S11).

**Fig. 9:**
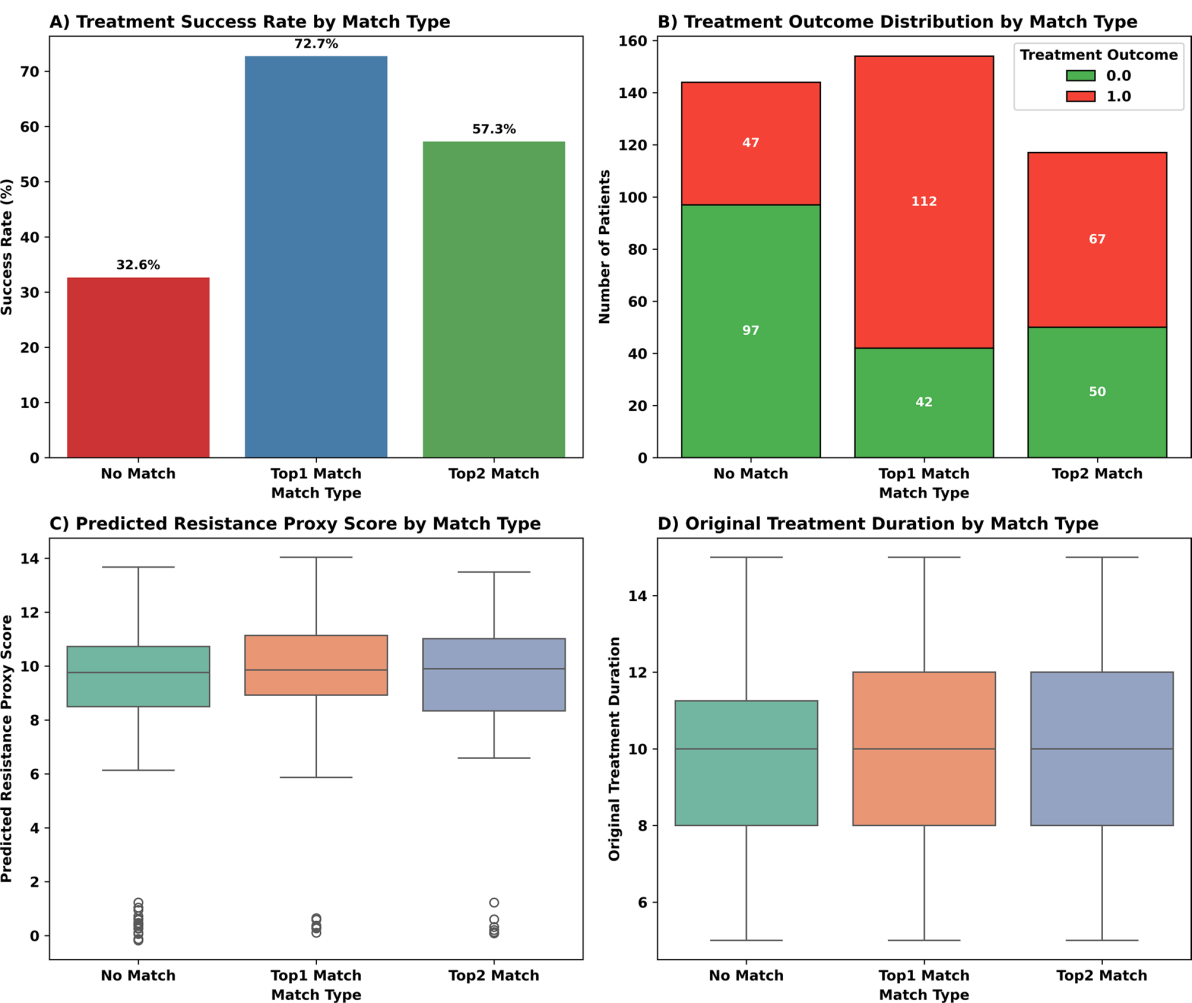
Match-based evaluation of treatment recommendation performance. (**A**) Treatment success rate by match type between the recommended drug and the actual prescribed drug. (**B**) Distribution of treatment outcomes across match categories shown as a stacked bar chart. (**C**) Predicted resistance proxy score stratified by match type. (**D**) Original treatment duration by match type. Together, these panels assess how agreement between model-recommended and prescribed treatments relates to treatment success, predicted resistance, and treatment duration

Stratified match-type analysis revealed meaningful differences in initial treatment outcomes. The Top-1 Match group exhibited the highest treatment success rate (72.73%), followed by the Top-2 Match group (57.26%), while the No Match group had the lowest success rate (32.64%). These findings indicate that alignment between the model’s primary recommendation and the drug prescribed in practice is associated with substantially higher treatment success. Interestingly, the No Match group had the lowest mean predicted resistance proxy score (8.63) compared with Top-1 Match (9.58) and Top-2 Match (9.32) groups. This suggests that the model may occasionally prioritize treatment options with lower predicted resistance risk even when those drugs differ from historical prescriptions (Table S5; Fig. 9A and B).

A crucial distinction in the interpretation of drug recommendation systems is brought to light by this evaluation: a departure from actual prescribing does not always signify model failure. Clinical prescriptions may reflect contextual considerations such as availability, clinician experience, or patient-specific factors that are not fully represented in the dataset. Therefore, discrepancies between model and physician choices may reflect alternative optimization priorities rather than simple misclassification.

The highest success rate was observed in the Top-1 Match group, confirming that the model frequently aligns with effective real-world prescriptions. Treatment success remained moderately high in the Top-2 Match group, suggesting that the model’s second-ranked recommendation may still represent a clinically viable alternative. Such near-miss predictions are particularly relevant in clinical decision support systems where multiple treatment options may be acceptable depending on contextual factors.

In contrast, the No Match group showed substantially poorer outcomes, reinforcing that disagreement between model recommendations and clinical prescriptions may correspond to more challenging treatment scenarios or cases where none of the model’s preferred options were selected. This group may therefore provide valuable insights for retrospective decision review, particularly when examining cases of treatment failure. These findings are consistent with emerging clinical AI research emphasizing that the value of decision-support systems should be evaluated not only through classification accuracy but also through their relationship with downstream patient outcomes and antimicrobial stewardship goals [23].

#### Confidence analysis

We conducted two confidence quantification assessments to evaluate the certainty of AI-generated drug recommendations: absolute confidence classification, which assessed the model’s anticipated success probability of the top-ranked drug (Top1_Probability) for each patient. The reccommendation was deemed Confident if this value was ≥ 0.5 (see calibration) or Ambiguous otherwise. The calibration above demonstrated that predictions at 0.4 were close to the calibration standard, and this threshold is consistent with the conventional practice of treating outputs above 0.5 as high-certainty in probabilistic binary classification [84] (Table S6).

**Fig. 10:**
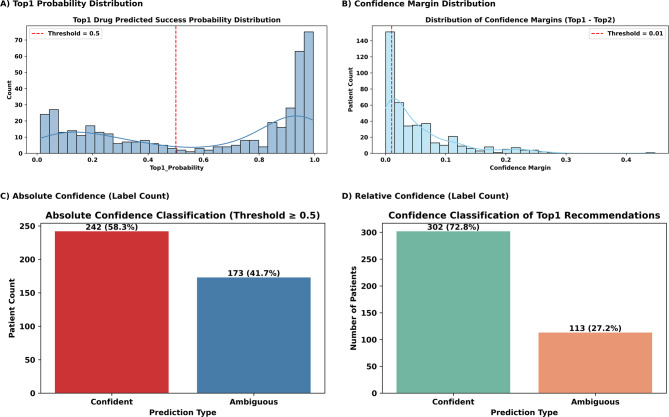
Confidence analysis of Top1 drug recommendations across 415 patients. The predicted success probabilities (**A**) show a bimodal distribution. The confidence margin distribution (**B**), defined as the difference between the Top1 and Top2 predicted probabilities, reveals a strong skew toward low margins. Panels (**C**) and (**D**) summarize the absolute (Top1_Probability ≥ 0.5) and relative (margin ≥ 0.01) confidence classifications, respectively

For Relative Confidence, we determined the confidence margin between the top two drug probabilities to evaluate model preference between medications that were closely ranked: Top1_Probability − Top2_Probability = Confidence Margin. In order to capture even little ranking dominance while being robust against prediction noise, a forecast was deemed confident if this margin was ≥ 0.01. There is a clear bimodal pattern in the Top1_Probability histogram (Fig. 10A). One distinct cluster of predictions is close to 1.0, which denotes great certainty, and another is close to zero. According to bar plots, 242 patients were classified as confident, while 173 patients were classified as ambiguous based on Top1 predicted probabilities (≥0.5), corresponding to 58.31% confident predictions under the absolute confidence criterion (Fig. 10C). With a strong tail skewed towards smaller margins, most Top1–Top2 margins fall below 0.05. To strike a balance between inclusivity and model confidence, a criterion of 0.01 was applied (Fig. 10B). Using this margin-based approach, 302 patients (72.77%) obtained confident predictions, while 113 patients (27.23%) were classified as ambiguous. This result demonstrates the advantage of employing a more sensitive threshold (0.01) for ranking clarity, as the relative confidence approach identifies a larger proportion of recommendations as confidently ranked compared with the absolute probability criterion (Fig. 10D).

#### Treatment discrepancy analysis

We conducted a treatment discrepancy analysis, which simulates and compares the expected success probabilities between (i) the model’s Top1 drug recommendation and (ii) the medication that the clinician actually prescribed, in order to assess the dependability and potential clinical utility of model-driven treatment recommendations.

Using the prediction output, we first looked for instances when the model’s Top1 recommendation deviated from the observed clinical prescription. From a held-out test set, which included encoded drug vectors for simulation, patient-level clinical and biochemical data were acquired. By re-introducing the patient record and switching the drug vector to activate the clinician-selected medication (1 for the actual drug, 0 for others), we were able to mimic the success probability of the actual prescribed drug using an outcome model. We were able to calculate ΔProbability = P (Model Top1) – P (Actual Prescribed) as a result. A positive Δ indicates that, according to the internal patterns of the model, the drug suggested by the model has a higher predicted probabilityof producing a successful course of therapy than the medication chosen by the doctor. Age, sex, inpatient duration, comorbidities, and pathogen-type metadata were added to a case-study subset of high-confidence differences (Δ > 0.05) to enhance interpretability and possible therapeutic translation.

**Fig. 11:**
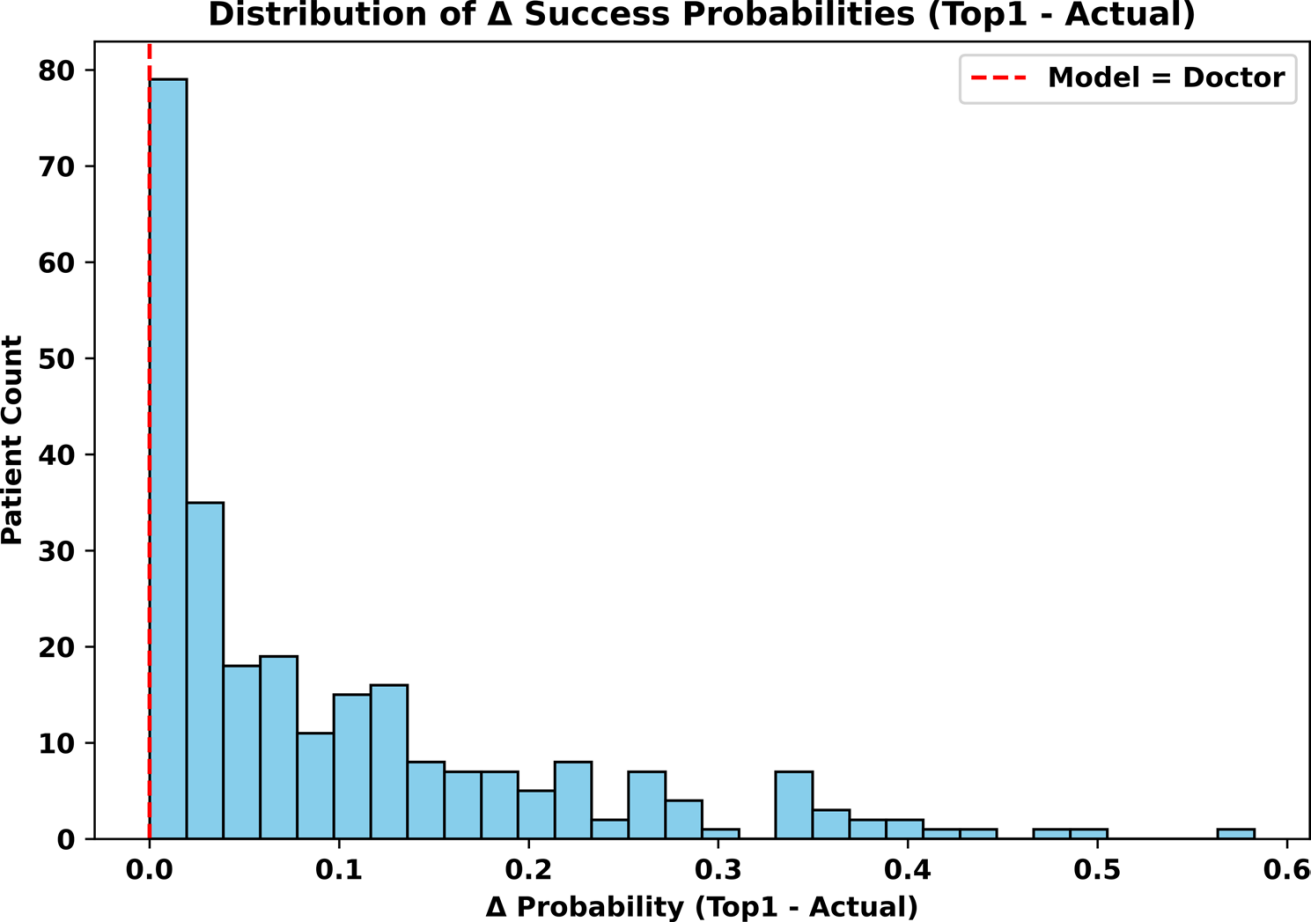
Distribution of Δ success probabilities (Top1-actual). Histogram illustrating the distribution of predicted success probability differences between the model’s Top1 drug recommendation and the clinician’s prescribed drug across 415 patients. A positive Δ value indicates higher predicted success for the model’s recommendation. The red dashed line (Δ = 0) represents cases where the model and clinician have equivalent predicted efficacy

In 261 (62.89%) of the 415 test patients, the model’s Top1 suggestion deviated from the medication provided by the clinician, resulting in inconsistent treatment choices. All discrepant cases (261/261, 100%) produced a positive Δ Probability, meaning that the model consistently projected higher success likelihoods for the drugs it recommended compared with the prescription recorded in the clinical data. The distribution of Δ values, which had a strong right-skew, is illustrated in Fig. 11. While many discrepancies were modest, a subset of cases showed substantial predicted improvements in treatment success probability, indicating that the model occasionally identified alternative drug options with markedly higher predicted efficacy. To further explore these cases, 10 high-confidence case studies (ΔProbability > 0.15) were selected for qualitative review (Table S7). These patients often exhibited clinical characteristics that may complicate treatment decisions, including variation in resistance proxy scores, patient demographics, or infection severity indicators.

When there is disagreement, the machine learning model regularly suggests therapies that are expected to function better than the clinician’s selection, at least according to success probability derived from training data, as this discrepancy study shows. This suggests a possible discrepancy between data-driven optimization and empirical decision-making, but it does not imply a clinical error. The potential of AI-guided medication selection in pharmacogenomics, cancer, and infectious illnesses has been highlighted by a number of research [23, 84–86]. Integrating probabilistic models provides a potent way to assist clinicians in the context of infectious disease, where patient variability and resistance patterns can significantly change outcomes, especially in environments with high levels of uncertainty or inadequate antimicrobial stewardship infrastructure.

A clinically interpretable proxy for confidence in switching to the model’s advice is the Δ Probability metric. The model’s calibration was acceptable for all drug classes, and the encoded patient-drug interaction captured meaningful predictive dynamics, although its dependability remains contingent on several assumptions. This framework assumes that unmeasured confounding factors, such as doctor intuition or contextual knowledge that is not recorded in features, are minimal. Therefore, before any therapeutic integration, prospective validation in real-world situations is essential, even though the model was statistically favored in 100% of discrepant cases. Future research should include mixed-methods prescriber feedback, causal inference frameworks, and uncertainty quantification.

#### Risk triage

We performed a stratified risk analysis based on the predicted antibiotic resistance proxy score generated by the machine learning framework. Patients were divided into four equally sized strata by partitioning resistance scores into quartiles, labeled Q1 (Lowest Risk) through Q4 (Highest Risk). For each quartile, we computed the mean, minimum, and maximum resistance proxy scores, as well as the number of patients within each group. This stratification enables examination of the progressive gradient of resistance risk across the population. The resulting quartile distribution demonstrates clear separation between patient risk groups. Patients in the lowest-risk quartile (Q1) exhibited a mean resistance proxy score of 5.20, with values ranging from −0.18 to 8.54 across 104 patients. In contrast, the highest-risk quartile (Q4) showed substantially elevated resistance scores, with a mean of 11.96 and a range from 10.96 to 14.04 across 104 patients. Intermediate quartiles (Q2 and Q3) displayed progressively increasing resistance levels, with mean scores of 9.27 and 10.30, respectively. This monotonic increase in resistance scores across quartiles indicates a consistent gradient of predicted antimicrobial resistance risk within the dataset (Table S8).

**Fig. 12:**
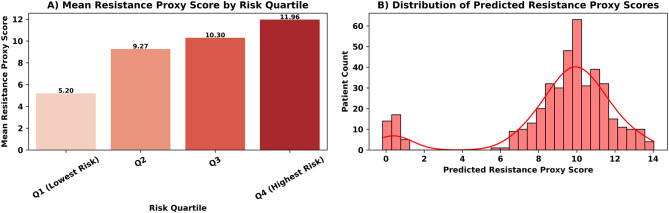
Resistance risk stratification across patient quartiles (Q1–Q4) based on predicted resistance proxy scores. Patients were grouped from lowest risk (Q1) to highest risk (Q4). (**A**) Mean resistance proxy score within each quartile. (**B**) Distribution of predicted resistance proxy scores across all patients

Risk stratification based on resistance likelihood provides a practical method for identifying patient groups that may require closer monitoring or more cautious antimicrobial selection. Patients in the highest-risk quartile represent clinically important profiles for intensified monitoring, as elevated resistance proxy scores may indicate infections more likely to involve resistant pathogens or reduced treatment responsiveness. Such cases may benefit from earlier susceptibility testing, broader-spectrum therapy, or enhanced clinical oversight. This stratified approach also supports antimicrobial stewardship efforts by enabling clinicians to prioritize resources toward patients at higher predicted resistance risk. Previous studies have shown that infections associated with antimicrobial resistance often require more complex treatment strategies and may be linked to poorer clinical outcomes [5, 87, 88]. By categorizing patients according to predicted resistance burden, clinicians can better anticipate treatment complexity and guide empirical antibiotic selection, particularly in resource-constrained healthcare settings where diagnostic infrastructure may be limited (Fig. 12).

#### Per drug summary analysis

**Fig. 13:**
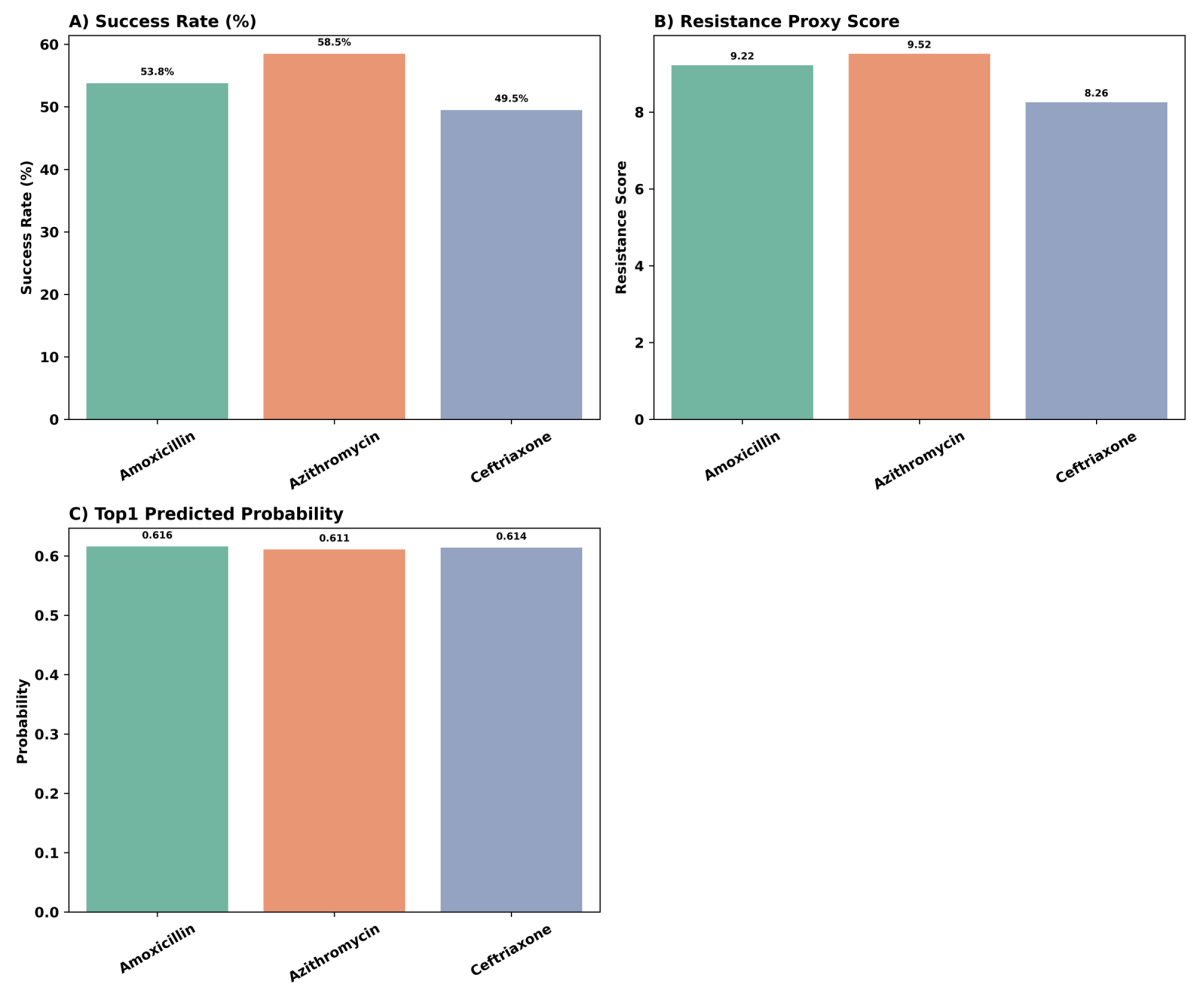
Presents a comparative grid of key indicators, including success rate (**A**), resistance proxy score (**B**), and Top1 probability per drug (**C**). These differences highlight potential optimization opportunities in antibiotic stewardship and treatment selection

Using predictions from our treatment outcome model, we conducted a per-drug stratified study to evaluate drug-specific performance and model interpretation. For summary data such as mean success rate, resistance proxy score, and Top1 likelihood (model confidence), patients were categorized according to the medications they actually received. Amoxicillin, azithromycin, and ceftriaxone had counts of 165, 127, and 123, respectively (Fig. 13 and Table S9). Each medicine had a different mean Top1 probability and treatment success rate. Azithromycin demonstrated the highest clinical success rate (62.99%) and the highest mean model confidence (Top1 probability = 0.622), indicating stronger predictive certainty for this treatment option. Amoxicillin showed a moderate success rate (52.73%) with a mean Top1 probability of 0.616, while ceftriaxone had the lowest success rate (47.97%) and lower model confidence (Top1 probability = 0.563). Resistance proxy scores also varied across the three drugs. Azithromycin had the highest mean resistance proxy score (9.51), followed by amoxicillin (9.07) and ceftriaxone (8.98). These differences highlight potential variability in predicted resistance patterns and therapeutic response across treatment options.

This analysis illustrates how SHAP and outcome stratification can be used to bridge the gap between model interpretability and clinical relevance. Antimicrobial resistance trends reported in typhoid treatment studies are partially reflected in the differing resistance proxy scores and predicted success rates across the evaluated drugs. Model confidence (Top1 probability) can serve as a quantitative indicator of prediction reliability, helping identify treatments where the model is more or less certain about expected outcomes. Individualized feature breakdowns enabled by SHAP integration can further support antimicrobial policy development and clinical decision support systems by clarifying how patient-specific factors influence treatment predictions.

We repeated the simulation process using the resistance model (minimum predicted score/value) in addition to the outcome model in order to further refine our simulation predictions. When the results were compared to those of the outcome model alone, it was found that they were identical. While internal validation demonstrated robust performance, external validation remains necessary to confirm generalizability across diverse clinical settings.

## Limitations, potential biases, and future directions

While the proposed framework demonstrates promising performance, it is important to emphasize that the current study is based on a single-cohort clinical dataset, with original patient records preserved and augmented by engineered and simulation-derived features generated at the feature level rather than through synthetic patient creation, and has not yet undergone external clinical validation; therefore, findings should be interpreted as exploratory decision-support insights rather than definitive treatment recommendations.

Several forms of bias may influence model behavior. These include selection bias arising from cohort construction, feature engineering bias introduced through rule-based derivation of variables such as resistance proxy scores, and representation bias due to under-sampling of rare but clinically important patient subgroups. Additionally, reliance on symptom-based and laboratory-derived features without microbiological confirmation may introduce measurement bias, particularly in distinguishing typhoid from other febrile illnesses. These biases may influence both model discrimination and calibration, particularly in underrepresented subgroups, potentially leading to over- or under-estimation of treatment benefit. This could translate into suboptimal antibiotic recommendations in edge-case patients if deployed without appropriate safeguards.

A key data-related limitation arises from the use of synthetic augmentation via nearest-neighbour sampling. While this approach preserves marginal feature distributions, it may not fully capture higher-order joint dependencies between variables. This is particularly relevant for rare clinical constellations such as co-occurring severe presentations, specific antibiotic exposures, and treatment failure, where even small deviations in joint structure can influence model behavior. Furthermore, binary thresholding of clinical variables, while pragmatic, may introduce discontinuities near decision boundaries, and PCA-derived components, although effective for dimensionality reduction, may obscure biologically meaningful correlations. Future work should explore context-aware generative models such as conditional tabular generative adversarial networks (CTGANs) or variational autoencoders conditioned on clinical subgroups to better preserve complex feature interactions. Integrating supervised or pathway-informed dimensionality reduction approaches may further improve biological interpretability.

Model-specific limitations also warrant consideration. We observed systematic overestimation in high-confidence probabilty regions despite overall acceptable calibration. Prior work suggests that post-hoc calibration techniques such as isotonic regression or Platt scaling can improve probabilistic reliability in gradient-boosted models, including XGBoost, and should be explored in future iterations. In addition, the models rely exclusively on observed variables, including symptoms, laboratory markers, and demographic features, and therefore do not account for unmeasured confounders such as comorbidities, pharmacokinetic variability, prescriber decision-making, and treatment adherence. The counterfactual simulation framework further assumes conditional exchangeability, namely that treatment groups are comparable after adjustment for observed features. This assumption is unlikely to fully hold in real-world clinical settings, and therefore the reported ΔProbability estimates should be interpreted as associational rather than causal. Future work should incorporate formal causal inference approaches, including directed acyclic graph (DAG)-guided adjustment, propensity score methods, or inverse probability weighting, alongside integration of laboratory-confirmed antimicrobial susceptibility data (e.g., AST or MIC) where available to improve causal validity [72, 89].

### Generalizability and external validity of the current study

The analysis is based on a single-center cohort and does not include external or temporal validation. As a result, model performance may be influenced by site-specific factors, including local epidemiological patterns, healthcare practices, and antimicrobial resistance (AMR) dynamics, all of which are known to vary substantially across geographic regions. In particular, typhoid-related resistance profiles differ across endemic settings, potentially limiting the direct transferability of the model-derived treatment recommendations. Furthermore, variation in patient demographics, comorbidity burden, diagnostic workflows, and data quality may affect model robustness when the framework is applied in new environments. The cross-sectional design also limits the ability of the framework to capture temporal disease trajectories and evolving resistance patterns. Consistent with TRIPOD-AI recommendations, these findings should therefore be interpreted as context-specific, and the framework should not be considered clinically deployable without independent validation. Prior to real-world implementation, rigorous external validation, temporal validation, and prospective clinical evaluation will be required, alongside model updating, recalibration, and monitoring for data drift to ensure safe and reliable performance across heterogeneous patient populations [90].

Future work should prioritize external validation using independent datasets from diverse geographic and clinical settings, including multi-center cohorts where available. Temporal validation using prospectively collected data will also be essential to assess model stability under evolving resistance patterns. Where direct validation is not immediately feasible, model transportability may be improved through domain adaptation strategies, local recalibration, and periodic updating.

## Conclusion

This study demonstrates that machine-learning models can meaningfully support antibiotic selection in typhoid fever by leveraging routinely collected clinical data and patient-level phenotypic profiles. Across multiple predictive tasks, the framework identified individualized treatment strategies that were frequently associated with higher predicted success probabilities than the clinician-prescribed regimens recorded in the dataset. These findings do not imply clinical error; rather, they illustrate how data-driven optimization can complement empirical prescribing by highlighting situations where alternative therapies might merit consideration. The ΔProbability metric, in particular, offers a practical way to quantify potential improvements from drug switching, although its interpretation must remain associational until validated prospectively.

The framework underscores the potential of AI-guided prescribing to enhance antimicrobial stewardship, particularly in settings with constrained resources and heterogeneous patient presentations. Nonetheless, the present system remains exploratory. Translation to clinical practice will require external validation across diverse epidemiological settings, integration with microbiological and genomic resistance data, and mixed-methods engagement with frontline clinicians to ensure interpretability, usability, and implementation safety. With these steps, machine-learning approaches could evolve into reliable decision-support tools that augment, rather than replace, clinical expertise in infectious diseases management.

## Electronic supplementary material

Below is the link to the electronic supplementary material.

Supplementary Material 1

Supplementary Material 2

## Data Availability

The datasets analysed during the current study are publicly available from the GitHub repository: https://github.com/SsemuyigaMHC/TyphoidRx. All code and analytical pipelines used in this study are available in the same repository.

## References

[1] Buzilă ER, Dorneanu OS, Trofin F, Sima CM, Iancu LS. Assessing *Salmonella Typhi* pathogenicity and prevention: the crucial role of vaccination in combating typhoid fever. Int J Mol Sci. 2025;26(9):3981. https://www.mdpi.com/1422-0067/26/9/3981/htm. 10.3390/ijms26093981 PMC12071698 40362220

[2] Boakye Okyere P, Twumasi-Ankrah S, Newton S, Nkansah Darko S, Owusu Ansah M, Darko E, et al. Risk factors for typhoid fever: systematic review. JMIR Public Health Surveill. 2025;11(1):e67544. 10.2196/67544. 10.2196/67544 PMC12426575 40875987

[3] Parry CM, Hien TT, Dougan G, White NJ, Farrar JJ. Typhoid fever. N Engl J Med. 2002;347(22):1770–82. https://pubmed.ncbi.nlm.nih.gov/12456854/. 12456854 10.1056/NEJMra020201

[4] Crump JA, Mintz ED. Global trends in typhoid and paratyphoid fever. Clin Infect Dis. 2010;50(2):241–46. https://pubmed.ncbi.nlm.nih.gov/20014951/. 20014951 10.1086/649541 PMC2798017

[5] Ssemuyiga C. In-vitro determination of antibacterial activity of cannabis sativa against staphylococcus aureus. 2019. http://dissertations.mak.ac.ug/handle/20.500.12281/7027.

[6] Zhang X, Zhang D, Zhang X, Zhang X. Artificial intelligence applications in the diagnosis and treatment of bacterial infections. Front Microbiol. 2024;15:1449844. https://pmc.ncbi.nlm.nih.gov/articles/PMC11334354/. 39165576 10.3389/fmicb.2024.1449844 PMC11334354

[7] Charles S, Mahapatra RK. Artificial intelligence based de-novo design for novel Plasmodium falciparum plasmepsin (PM) X inhibitors. J Biomol Struct Dyn. 2023;1–16. https://www.tandfonline.com/doi/abs/10.1080/07391102.2023.2279700. 10.1080/07391102.2023.2279700 37943000

[8] Awe OI, Obura H, Ssemuyiga C, Mudibo E, Mwanga MJ. Enhanced deep convolutional neural network for SARS-CoV-2 variants classification. Front Artif Intell. 2025;8:1512003. 10.3389/frai.2025.1512003. 40988919 10.3389/frai.2025.1512003 PMC12450893

[9] Klemm EJ, Shakoor S, Page AJ, Qamar FN, Judge K, Saeed DK, et al. Emergence of an extensively drug-resistant *Salmonella enterica* serovar Typhi clone harboring a promiscuous plasmid encoding resistance to fluoroquinolones and third-generation cephalosporins. mBio. 2018;9(1). 10.1128/mBio.00105-18. 10.1128/mBio.00105-18 PMC5821095 29463654

[10] Pennisi F, Pinto A, Ricciardi GE, Signorelli C, Gianfredi V. The role of artificial intelligence and machine learning models in antimicrobial stewardship in public health: a narrative review. Antibiotics. 2025;14(2):134. https://www.mdpi.com/2079-6382/14/2/134/htm. 40001378 10.3390/antibiotics14020134 PMC11851606

[11] Alami H, Lehoux P, Denis JL, Motulsky A, Petitgand C, Savoldelli M, et al. Organizational readiness for artificial intelligence in health care: insights for decision-making and practice. J Health Organ Manag. 2021;35(1):106–14. 10.1108/JHOM-03-2020-0074. 10.1108/JHOM-03-2020-0074 33258359

[12] de la Lastra JMP, Wardell SJT, Pal T, de la Fuente-Nunez C, Pletzer D. From data to decisions: leveraging artificial intelligence and machine learning in combating antimicrobial resistance – a comprehensive review. J Med Syst. 2024;48(1):1–14. 10.1007/s10916-024-02089-5. 10.1007/s10916-024-02089-5 PMC11294375 39088151

[13] Algain S, Marra AR, Kobayashi T, Marra PS, Celeghini PD, Hsieh MK, et al. Can we rely on artificial intelligence to guide antimicrobial therapy? A systematic literature review. Antimicrob Steward Healthc Epidemiol. 2025;5(1):e90. https://www.cambridge.org/core/journals/antimicrobial-stewardship-and-healthcare-epidemiology/article/can-we-rely-on-artificial-intelligence-to-guide-antimicrobial-therapy-a-systematic-literature-review/8239BEF5A37E8747203593A2D6C99DAE. 10.1017/ash.2025.47 PMC11986881 40226293

[14] Maviglia R, Michi T, Passaro D, Raggi V, Bocci MG, Piervincenzi E, et al. Machine learning and antibiotic management. Antibiotics. 2022;11(3):304. https://www.mdpi.com/2079-6382/11/3/304/htm. 35326768 10.3390/antibiotics11030304 PMC8944459

[15] Ribers MA, Ullrich H, Arpi RM, Bjerrum L, Cristina G, Currea C, et al. Battling antibiotic resistance: can machine learning improve prescribing? SSRN Electron J. 2019. https://arxiv.org/pdf/1906.03044.

[16] Revell AD, Wang D, Wood R, Morrow C, Tempelman H, Hamers RL, et al. An update to the HIV-TRePS system: the development and evaluation of new global and local computational models to predict HIV treatment outcomes, with or without a genotype. J Antimicrob Chemother. 2016;71(10):2928. https://pmc.ncbi.nlm.nih.gov/articles/PMC5031919/. 27330070 10.1093/jac/dkw217 PMC5031919

[17] Attai K, Ekpenyong M, Amannah C, Asuquo D, Ajuga P, Obot O, et al. Enhancing the interpretability of malaria and typhoid diagnosis with explainable ai and large language models. Trop Med Infect Dis. 2024;9(9):216. 10.3390/tropicalmed9090216. 10.3390/tropicalmed9090216 PMC11436130 39330905

[18] Amannah C, Attai KF, Uzoka FM. A data-driven intelligent methodology for developing explainable diagnostic model for febrile diseases. Algorithms. 2025;18(4):190. 10.3390/a18040190. 32607472 10.1038/s42256-019-0138-9 PMC7326367

[19] Asuquo D, Attai K, Obot O, Ekpenyong M, Akwaowo C, Arnold K, et al. Febrile disease modeling and diagnosis system for optimizing medical decisions in resource-scarce settings. Clinical eHealth. 2024;7(1):52–76. 10.1016/j.ceh.2024.05.001. 30943338 10.1056/NEJMra1814259

[20] Masum AKM, Khan MFI, Hassan MM, Bitto AK, Farid DM, Rahman T. Enhancing dengue fever diagnosis: a machine learning framework with stacking ensemble and shap explainability. 2025 IEEE International Conference on Quantum Photonics, Artificial Intelligence, and Networking, QPAIN 2025. 2025. 10.1109/QPAIN66474.2025.11171651.

[21] Chen T, Guestrin C. XG Boost: a scalable tree boosting system. Proceedings of the ACM SIGKDD International Conference on Knowledge Discovery and Data Mining. 2016;13-17:785–94. 10.1145/2939672.2939785/SUPPL_FILE/KDD2016_CHEN_BOOSTING_SYSTEM_01-ACM.MP4. 22751760 10.7326/0003-4819-157-1-201206190-00429

[22] Lundberg SM, Erion G, Chen H, Degrave A, Prutkin JM, Nair B, et al. From local explanations to global understanding with explainable AI for trees. Nat Mach Intell. 2020;2(1):56–67. 10.1038/s42256-019-0138-9. 10.1038/s42256-019-0138-9 PMC7326367 32607472

[23] Rajkomar A, Dean J, Medicine IKEJ of, 2019 undefined. Machine learning in medicine. N Engl J Med. 2019;380(14):1347–58. 10.1056/NEJMRA1814259. PubMed PMID: 30943338. 10.1056/NEJMra1814259 30943338

[24] Shickel, B, Tighe PJ, Bihorac A, Rashidi P. Deep EHR: a survey of recent advances in deep learning techniques for electronic health record (EHR) analysis. IEEE J. Biomed. Health Inform. 2018;22:1589–604. 10.1109/JBHI.2017.2767063. 10.1109/JBHI.2017.2767063 PMC6043423 29989977

[25] Carson JL, Grossman BJ, Kleinman S, Tinmouth AT, Marques MB, Fung MK, et al. Red blood cell transfusion: a clinical practice guideline from the AABB. Ann Intern Med. 2012;157(1):49–58. 10.7326/0003-4819-157-1-201206190-00429. PubMed PMID: 22751760. 10.7326/0003-4819-157-1-201206190-00429 22751760

[26] Platelet Count (PLT). Normal range, test results & meaning [Internet]. [cited 2025 May 3]. Available from: https://my.clevelandclinic.org/health/diagnostics/21782-platelet-count.

[27] Sadiq NM, Anastasopoulou C, Patel G, Badireddy M. Hypercalcemia. Stat Pearls. 2024. PubMed PMID: 28613465. 28613465

[28] Schafer AL, Shoback DM. Hypocalcemia: diagnosis and treatment. Endotext. 2016. PubMed PMID: 25905251.

[29] Calcium Blood Test: what It Is & Results [Internet]. [cited 2025 May 3]. Available from: https://my.clevelandclinic.org/health/diagnostics/22021-calcium-blood-test 32939066 10.1038/s41586-020-2649-2 PMC7759461

[30] Rastegar A. Serum Potassium. BMJ. 1990;2(4743):1341–2. 10.1136/bmj.2.4743.1341-c. PubMed PMID: 21250149.

[31] Potassium Levels Blood Test: high vs. Low, Normal K Level [Internet]. [cited 2025 May 3]. Available from: https://www.webmd.com/a-to-z-guides/potassium-blood-test.

[32] Typhoid Fever - Symptoms, treatment, causes and diagnosis [Internet].[cited 2025 May 9]. Available from: https://www.careinsurance.com/blog/health-insurance-articles/typhoid-how-dangerous-it-could-be?utm_source=chatgpt.com.

[33] Harris CR, Jarrod Millman K, vander Walt SJ, Gommers R, Virtanen P, Cournapeau D, et al. Array programming with NumPy. Nature. 2020;585:357. 10.1038/s41586-020-2649-2. 10.1038/s41586-020-2649-2 PMC7759461 32939066

[34] SciPy WM. 2010. Data structures for statistical computing in Python. 2010 [cited 2025 Jul 9]. Available from: https://pdfs.semanticscholar.org/ef4e/f7f38bb907e5d7b4df3e6ff1db269d4970f5.pdf.

[35] Pedregosa F, Michel V, Grisel O, Blondel M, Prettenhofer P, Weiss R, et al. Scikit-learn: machine learning in python. J Mach Learn Res [Internet]. 2011 [cited 2025 May 9];12(85):2825–30. Available from: http://jmlr.org/papers/v12/pedregosa11a.html.

[36] Chawla NV, Bowyer KW, Hall LO, Kegelmeyer WP. SMOTE: synthetic minority over-sampling technique. Artif Intell Res. 2002;16:321–57. 10.1613/JAIR.953.

[37] McInnes L, Healy J, Melville J. UMAP: uniform manifold approximation and projection for dimension reduction [Internet]. 2018 [cited 2025 Jul 7]. Available from: https://arxiv.org/pdf/1802.03426.

[38] Maaten L Van der, research GHJ of machine learning, 2008 undefined. Visualizing data using t-SNE. jmlr.org [Internet].2008 [cited 2025 Jul 7];9:2579–605. Available from: https://www.jmlr.org/papers/volume9/vandermaaten08a/vandermaaten08a.pdf?fbcl.

[39] Lundberg S, information SL A in neural, 2017 undefined. A unified approach to interpreting model predictions. Advances in neural information processing systems. 2017. [Internet]. [cited2025 Jul 9]. Available from: https://proceedings.neurips.cc/paper/2017/hash/8a20a8621978632d76c43dfd28b67767-Abstract.html.

[40] Hunter JD. Matplotlib: a 2D graphics environment. Comput Sci Eng. 2007;9(3):90–5. 10.1109/MCSE.2007.55.

[41] Software MWJ. Seaborn: statistical data visualization. J Open Source Softw. 2021. 10.21105/JOSS.03021.PDF.

[42] Flexible imputation of missing data. Second edition. | Stef van Buuren [Internet]. [cited 2025 May 7]. Available from: https://stefvanbuuren.name/publication/vanbuuren-2018/. 26603918 10.1016/S0140-6736(15)00474-2

[43] Beam AL, Kohane IS. Big data and machine learning in health care. JAMA. 2018;319(13):1317–8. 10.1001/jama.2017.18391 PubMed PMID: 29532063. 29532063 10.1001/jama.2017.18391

[44] Johnson AEW, Pollard TJ, Shen L, Lehman LWH, Feng M, Ghassemi M, et al. Data Descriptor: MIMIC-III, a freely accessible critical care database. Sci Data. 2016;3(1):160035. 10.1038/sdata.2016.35. 27219127 10.1038/sdata.2016.35 PMC4878278

[45] Mogasale V, Maskery B, Ochiai RL, Lee JS, Mogasale V V., Ramani E, et al. Burden of typhoid fever in low-income and middle-income countries: asystematic, literature-based update with risk-factor adjustment. Lancet Glob Health. 2014;2(10):e570–80. 10.1016/S2214-109X(14)70301-8. PubMed PMID: 25304633. 10.1016/S2214-109X(14)70301-8 25304633

[46] Background document: the diagnosis, treatment and prevention of typhoid fever [Internet]. [cited 2025 May 7]. Available from: https://iris.who.int/handle/10665/370492. 31532961 10.1056/NEJMra1804281

[47] Dyson ZA, Klemm EJ, Palmer S, Dougan G. Antibiotic resistance and typhoid. Clin Infect Dis. 2019;68(Suppl 2):S165. 10.1093/CID/CIY1111 PubMed PMID: 30845331. 30845331 10.1093/cid/ciy1111 PMC6405283

[48] Laxminarayan R, Matsoso P, Pant S, Brower C, Røttingen JA, Klugman K, et al. Access to effective antimicrobials: a worldwide challenge. The Lancet. 2016;387(10014):168–75. 10.1016/S0140-6736(15)00474-2. PubMed PMID: 26603918. 10.1016/S0140-6736(15)00474-2 26603918

[49] Crump JA, Luby SP, Mintz ED. The global burden of typhoid fever. Bull World Health Organ. 2004;82(5):346. PubMed PMID: 15298225. 15298225 PMC2622843

[50] Hosoglu S, Geyik MF, Akalin S, Ayaz C, Kokoglu OF, Loeb M. A simple validated prediction rule to diagnose typhoid fever in Turkey. Trans R Soc Trop Med Hyg. 2006;100(11):1068–74. 10.1016/J.TRSTMH.2005.12.007. PubMed PMID: 16697432. 10.1016/j.trstmh.2005.12.007 16697432

[51] Biochemistry of Lipids, Lipoproteins and Membranes | ScienceDirect [Internet]. [cited 2025 May 8]. Available from: https://www.sciencedirect.com/book/9780444532190/biochemistry-of-lipids-lipoproteins-and-membranes.

[52] Ganz T. Anemia of Inflammation. N Engl J Med. 2019;381(12):1148–57. 10.1056/NEJMRA1804281. PubMed PMID: 31532961. 10.1056/NEJMra1804281 31532961

[53] Semenza GL. Oxygen sensing, homeostasis, and disease. N Engl J Med. 2011;365(6):537–47. 10.1056/NEJMRA1011165. PubMed PMID: 21830968. 10.1056/NEJMra1011165 21830968

[54] Charles S, Edgar MP, Kasoma NA. The hunt for antipox compounds against monkeypox virus thymidylate kinase and scaffolding protein leveraging pharmacophore modeling, molecular docking, ADMET studies and molecular dynamics simulation studies. Virology & Mycology. 2023;12(4):1–14. 10.35248/2161-0517.23.12.280. 28904005 10.1098/rsif.2017.0213 PMC5636267

[55] David CC, Jacobs DJ. Principal component analysis: a method for determining the essential dynamics of proteins. Methods Mol Biol. 2014;1084:193–226. 10.1007/978-1-62703-658-0_11. PubMed PMID: 24061923. 24061923 10.1007/978-1-62703-658-0_11 PMC4676806

[56] Wold S, Esbensen K, Geladi P. Principal component analysis. Chemom Intell Lab Syst. 1987;2(1–3):37–52. 10.1016/0169-7439(87)80084-9.

[57] Almadhoun MB, Burhanuddin M. Optimizing feature selection and machine learning algorithms for early detection of prediabetes risk: comparative study. JMIR Bioinform Biotech. 2025;6:e70621. 10.2196/70621. 10.2196/70621 PMC12314567 41342190

[58] Varma S, Simon R. Bias in error estimation when using cross-validation for model selection. BMC Bioinform. 2006;7(1):91. 10.1186/1471-2105-7-91. PubMed PMID: 16504092. 10.1186/1471-2105-7-91 PMC1397873 16504092

[59] Bergstra J, Ca JB, Ca YB. Random search for hyper-parameter optimization. J Mach Learn Res. 2012;13:281–305. 10.5555/2188385.2188395. 9620400 10.1128/jcm.36.6.1683-1687.1998 PMC104900

[60] Nakagawa S, Johnson PCD, Schielzeth H. The coefficient of determination R2 and intra-class correlation coefficient from generalized linear mixed-effects models revisited and expanded. J R Soc Interface. 2017;14(134). 10.1098/RSIF.2017.0213. PubMed PMID: 28904005. 10.1098/rsif.2017.0213 PMC5636267 28904005

[61] Krstajic D, Buturovic LJ, Leahy DE, Thomas S. Cross-validation pitfalls when selecting and assessing regression and classification models. J Cheminform. 2014;6(1):10. 10.1186/1758-2946-6-10. PubMed PMID: 24678909. 10.1186/1758-2946-6-10 PMC3994246 24678909

[62] Tibshirani RJ, Tibshirani R. A bias correction for the minimum error rate in cross-validation. 2009;3(2):822–9. 10.1214/08-AOAS224. 30842888 10.1007/s41664-018-0068-2 PMC6373628

[63] Parry CM, Vinh H, Chinh NT, Wain J, Campbell JI, Hien TT, et al. The influence of reduced susceptibility to fluoroquinolones in Salmonella enterica serovar Typhi on the clinical response to of loxacin therapy. PLoS Negl Trop Dis.2011;5(6). 10.1371/journal.pntd.0001163. PubMed PMID: 21713025. 10.1371/journal.pntd.0001163 PMC3119645 21713025

[64] Marchello C, Birkhold M. Complications and mortality of typhoid fever: a global systematic review and meta-analysis. Elsevier [Internet]. [cited 2025 Jul 7]. Available from: https://www.sciencedirect.com/science/article/pii/S0163445320306903. 10.1016/j.jinf.2020.10.030 PMC7754788 33144193

[65] Wain J, Song Diep T, Anh VH, Walsh AM, Thi Tuyet Hoa N, Parry CM, et al. Quantitation of bacteria in blood of typhoid fever patients and relationship between counts and clinical features, transmissibility, and antibiotic resistance. J Clin Microbiol [Internet]. 1998. Available from: https://journals.asm.org/journal/jcm. 10.1128/jcm.36.6.1683-1687.1998 PMC104900 9620400

[66] Levine M, Black R, Ferreccio C, Germanier R, Committee CT. Large-scale field trial of Ty21a live oral typhoid vaccine in enteric-coated capsule formulation. The Lancet. 1987. Elsevier [Internet]. [cited2025 Jul 7]. Available from: https://www.sciencedirect.com/science/article/pii/S0140673687904806. 2883393 10.1016/s0140-6736(87)90480-6

[67] Charles S, Pius Edgar M, Mahapatra RK. Host-directed anti-fusion aptamers and small molecules as respiratory syncytial virus (RSV) Inhibitors: an in silico-based study. J Biotechnol Biomed. 2024;7:485–97. 10.26502/jbb.2642-91280171.

[68] Xu Y, Goodacre R. On splitting training and validation set: a comparative study of cross-validation, bootstrap and systematic sampling for estimating the generalization performance of supervised learning. J Anal Test. 2018;2(3):249–62. 10.1007/S41664-018-0068-2. 10.1007/s41664-018-0068-2 PMC6373628 30842888

[69] Efron,B, Tibshirani RJ. An Introduction - Google Scholar [Internet]. 1993 [cited 2025 Jul 7]. Available from: https://scholar.google.com/scholar?hl=en%26as_sdt=0%2C5%26q=%E2%80%A2%09Efron%2C+B.%2C+%26+Tibshirani%2C+R.+J.+%281993%29.+An+Introduction+to+the+Bootstrap.+Chapman+%26+Hall%2FCRC.%26btnG=. 25411322 10.1177/0962280214558972 PMC5394463

[70] Antillón M, Warren JL, Crawford FW, Weinberger DM, Kürüm E, Pak GD, et al. The burden of typhoid fever in low- and middle-income countries: A meta-regression approach. PLoS Negl Trop Dis. 2017;11(2):e0005376. 10.1371/journal.pntd.0005376. PMID: 28241011; PMCID: PMC5344533. 10.1371/journal.pntd.0005376 PMC5344533 28241011

[71] Van Calster B, McLernon DJ, Van Smeden M, Wynants L, Steyerberg EW, Bossuyt P, et al. Calibration: the achilles heel of predictive analytics. BMC Med. 2019;17(1):1–7. 10.1186/S12916-019-1466-7/TABLES/1. PubMed PMID: 31842878. 10.1186/s12916-019-1466-7 PMC6912996 31842878

[72] Niculescu-Mizil A, Caruana RC. Predicting good probabilities with supervised learning. dl.acm.org. 2005;625–32. 10.1145/1102351.1102430. 30792131 10.1016/S1473-3099(18)30685-6 PMC6437314

[73] BRIER GW. Verification of forecasts expressed in terms of probability. Monthly weather review. 1950. 10.1175/1520-0493(1950)078. 30974881 10.3390/medicina55040099 PMC6524067

[74] Austin PC, Steyerberg EW. Events per variable (EPV) and the relative performance of different strategies for estimating the out-of-sample validity of logistic regression models. Stat Methods Med Res. 2017;26(2):796–808. 10.1177/0962280214558972. Epub 2014 Nov 19. PMID: 25411322. 10.1177/0962280214558972 PMC5394463 25411322

[75] Molnar C. Interpretable machine learning [Internet]. 2020 [cited2025 Jul 7]. Available from: https://books.google.com/books?hl=en%26lr=%26id=jBm3DwAAQBAJ%26oi=fnd%26pg=PP1%26dq=3.%09Molnar,+C.+(2022).+Interpretable+Machine+Learning:+A+Guide+for+Making+Black+Box+Models+Explainable+(2nd+ed.).+https://christophm.github.io/interpretable-ml-book/%26ots=EhwRUiFKY5%26sig=HgXrqnPQjLQtOWN-3zq6lRcfsc0. 15767266 10.1136/bmj.38398.500764.8F PMC555881

[76] Stanaway JD, Reiner RC, Blacker BF, Goldberg EM, Khalil IA, Troeger CE, et al. The global burden of typhoid and paratyphoid fevers: a systematic analysis for the Global Burden of Disease Study 2017. Lancet Infect Dis. 2019;19(4):369–81. 10.1016/S1473-3099(18)30685-6. PubMed PMID: 30792131. 10.1016/S1473-3099(18)30685-6 PMC6437314 30792131

[77] Tamelytė E, Vaičekauskienė G, Dagys A, Lapinskas T, Jankauskaitė L. Early blood biomarkers to improve sepsis/bacteremia diagnostics in pediatric emergency settings. Medicina. 2019;55(4):99. 10.3390/MEDICINA55040099. PubMed PMID: 30974881. 10.3390/medicina55040099 PMC6524067 30974881

[78] Ahmad M, Eckert C, ACM. Interpretable machine learning in healthcare. Proceedings of the 9th ACM international Conference on Bioinformatics, Computational Biology, and Health Informatics. 2018. 559–60. 10.1145/3233547.3233667.

[79] Attai KF, Amannah C, Ekpenyong M, Asuquo DE, Akputu OK, Obot OU, et al. Developing an explainable artificial intelligence system for the mobile-based diagnosis of febrile diseases using random forest, LIME, and GPT. Health Inform Res. 2025;31(2):125–35. 10.4258/hir.2025.31.2.125. 10.4258/hir.2025.31.2.125 PMC12086442 40384064

[80] Attai KF, Amannah C, EkpenyongM, Baadel S, Obot O, Asuquo D, et al. Predicting predisposition to tropical diseases in female adults using risk factors: an explainable-machine learning approach. Information. 2025;16(7):520. 10.3390/info16070520.

[81] Kawamoto K, Houlihan CA, Balas EA, Lobach DF. Improving clinical practice using clinical decision support systems: a systematic review oftrials to identify features critical to success. BMJ. 2005;330(7494):765. 10.1136/BMJ.38398.500764.8F. PubMed PMID: 15767266. 10.1136/bmj.38398.500764.8F PMC555881 15767266

[82] Shortliffe EH, Cimino JJ. Biomedical informatics: computer applications in health care and biomedicine: Fourth edition. Biomedical Informatics: Computer Applications in Health Care and Biomedicine: Fourth Edition. 2014;1–965. 10.1007/978-1-4471-4474-8/COVER.

[83] Jiang H, Kim Google Brain B, Guan MY, Gupta Google Research M. To trust or not to trust a classifier. Advances in neural information processing systems. 2018. [cited 2025 Jul 12]. Available from: https://proceedings.neurips.cc/paper/2018/hash/7180cffd6a8e829dacfc2a31b3f72ece-Abstract.html.

[84] Topol, E. (2019). Deep Medicine: How Artificial Intellige... - Google Scholar [Internet]. [cited2025 Jul 12]. Available from: https://scholar.google.com/scholar?hl=en%26as_sdt=0%2C5%26q=Topol%2C+E.+%282019%29.+Deep+Medicine%3A+How+Artificial+Intelligence+Can+Make+Healthcare+Human+Again.+Basic+Books.%26btnG=.

[85] Charles S, Edgar MP. Geometric deep learning prioritization and validation of cannabis phytochemicals as anti-hcv non-nucleoside direct-acting inhibitors. Biomed Eng Comput Biol. 2024;15. 10.1177/11795972241306881. 39678171 10.1177/11795972241306881 PMC11638990

[86] Charles S, Edgar MP, Mahapatra RK. Artificial intelligence based virtual screening study for competitive and allosteric inhibitors of the SARS-CoV-2 mainprotease. J Biomol Struct Dyn. 2023. 10.1080/07391102.2023.21884190. PubMed PMID: 36943715. 10.1080/07391102.2023.2188419 36943715

[87] Dellit TH, Owens RC, Mcgowan JE, Gerding DN, Weinstein RA, Burke JP, et al. Infectious diseases society of america and the society for healthcare epidemiology of america guidelines for developing an institutional program to enhance. Clinical infectious diseases. 2007. [cited 2025 Jul 13]. Available from: https://academic.oup.com/cid/article-abstract/44/2/159/328413. 10.1086/510393 17173212

[88] Spellberg B, Guidos R, Gilbert D, Bradley J, Boucher HW, Scheld WM, et al. The epidemic of antibiotic-resistant infections: a call to action for the medical community from the Infectious Diseases Society of America. Clinical infectious diseases. 2008. 10.1086/524891. 10.1086/524891 18171244

[89] Ojeda FM, Jansen ML, Thiéry A, Blankenberg S, Weimar C, Schmid M, et al. Calibrating machine learning approaches for probability estimation: a comprehensive comparison. Stat Med. 2023;42(29):5451–78. 10.1002/SIM.9921. PubMed PMID: 37849356. 10.1002/sim.9921 37849356

[90] Tyralis H, Papacharalampous G. A review of predictive uncertainty estimation with machine learning. Artif Intell Rev. 2024;57(4):1–65. 10.1007/S10462-023-10698-8/FIGURES/1.

