# Supplementary material for: Machine learning-driven decision support for antibiotic optimization in typhoid fever based on patient profiles
Source: BMC Med Inform Decis Mak. 2026 May 12;26:241. doi: 10.1186/s12911-026-03528-8 (PMC13335061; doi:10.1186/s12911-026-03528-8)
Supplement: Supplementary file 1 — Supplementary Material 1 [file 12911_2026_3528_MOESM1_ESM.docx]

**Machine Learning–Driven Drug Optimization for Typhoid Fever Based on Patient Profiles**

**Ssemuyiga Charles^1,2*^, Elminah Saru^3^, and Yusuf Abbas Aleshinloye^4^.**

**(**[**ssemuyigacharles11@gmail.com**](mailto:ssemuyigacharles11@gmail.com)**)**

^1^PharmaQsar Bioinformatics Firm, Kampala, Uganda. ^2^Department of Public Health, School of Public Health, Kampala International University, Kampala, Uganda. ^3^Department of Biochemistry and Biotechnology, Pwani University, Kenya. ^4^School of Mathematics and Computing, Kampala International University, Kampala, Uganda.

**
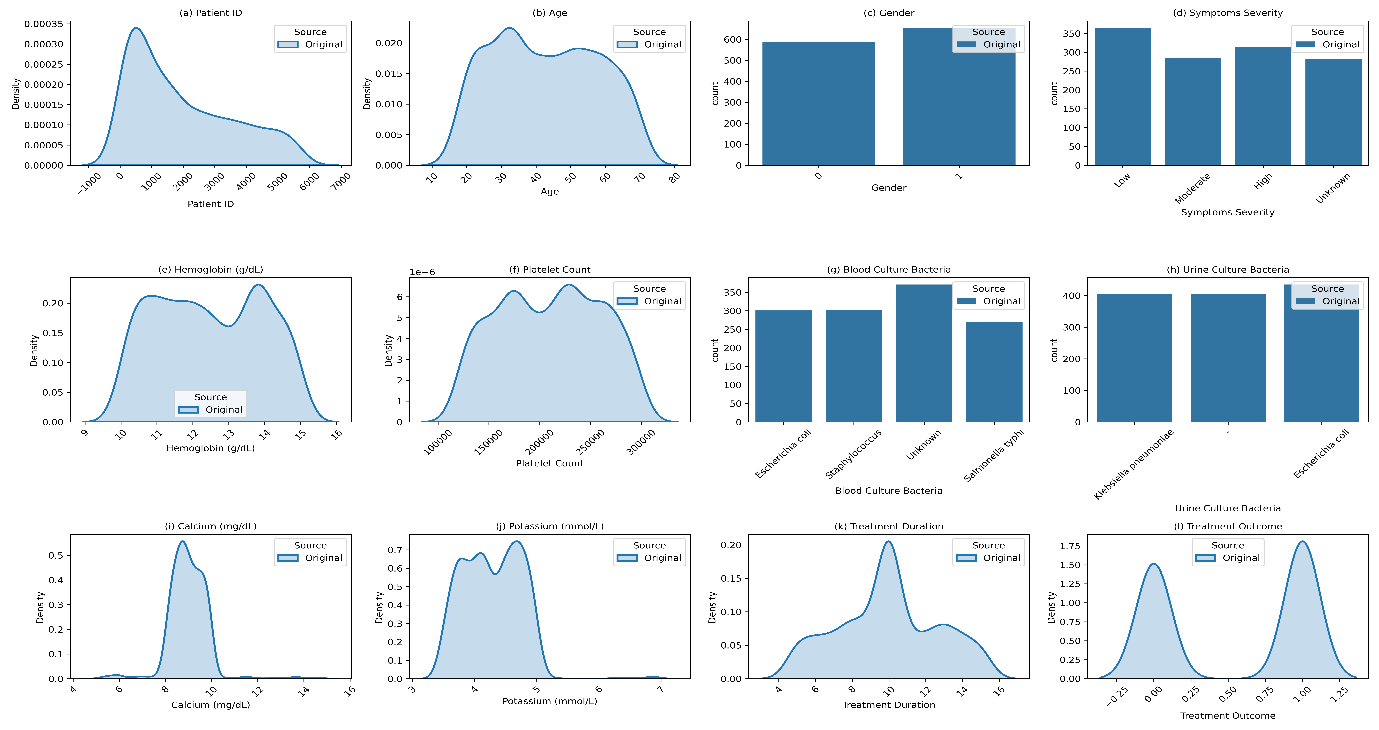
**

Figure S1: Variable distributions across the study cohort, highlighting dataset heterogeneity that underpins model specification and covariate adjustment.


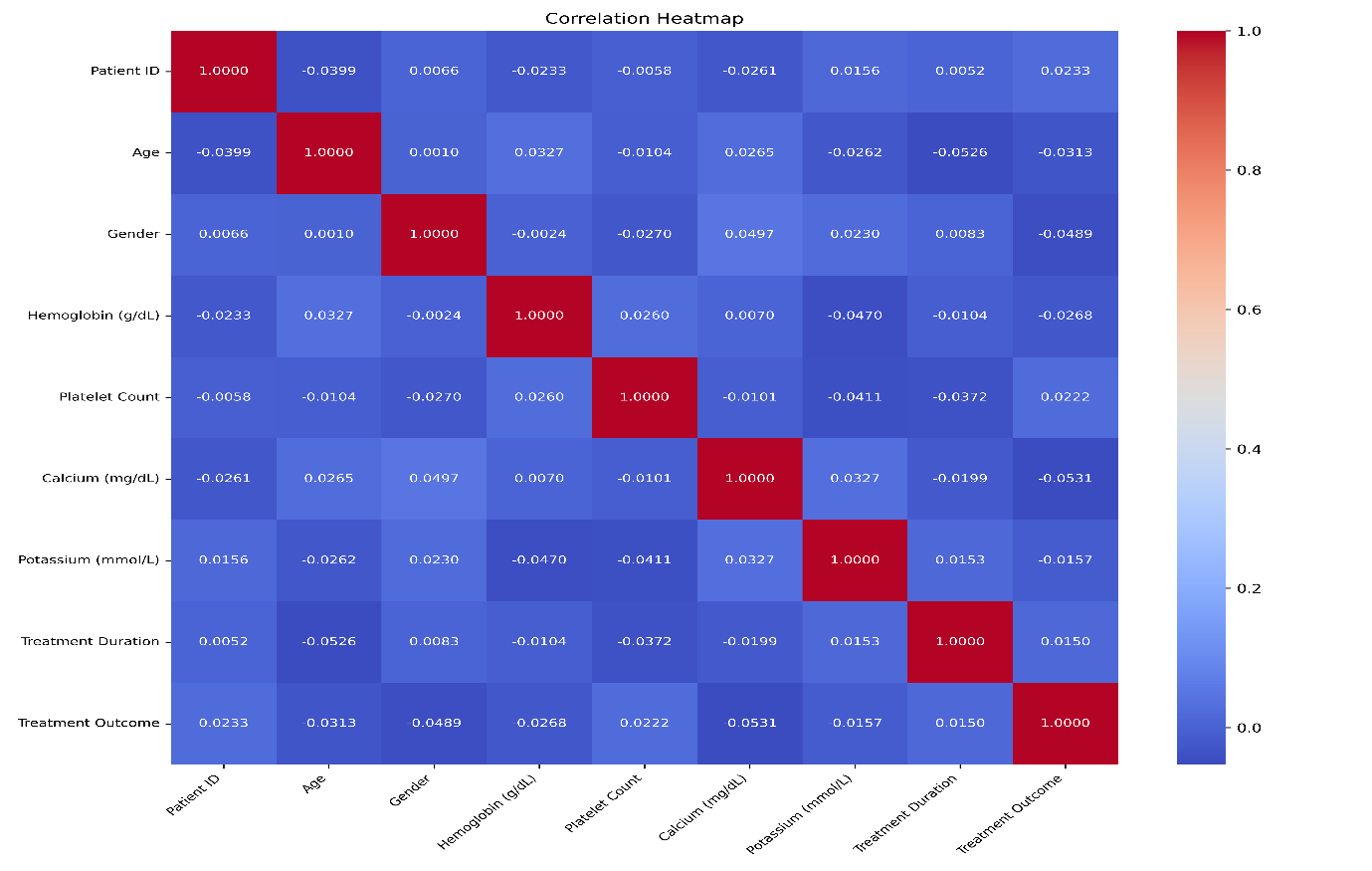


Figure S2: A heatmap showing Pearson correlation coefficients between numeric features in the dataset. The low-to-moderate correlations suggest minimal multicollinearity, ensuring that each variable may independently contribute to treatment outcome prediction.

Table S1: Summary of engineered features derived during preprocessing and simulation. The table describes variable types, encoding logic, and clinical relevance for each transformed or constructed feature used in the final machine learning models. These features reflect symptom severity, culture results, treatment factors, biochemical statuses, simulated metabolic disturbances, and principal components capturing latent patterns.

| **Feature Name** | **Type** | **Description** |
| --- | --- | --- |
| Severity_Score | Ordinal (1–3) | Numerical encoding of symptom severity: Low = 1, Moderate = 2, High = 3 |
| Culture_Concordance | Binary | 1 if the same organism is in both the blood and urine cultures; 0 otherwise |
| Salmonella_Presence | Binary | 1 if *Salmonella typhi* is detected in either blood or urine culture |
| Culture_Type | Categorical | 0 = No growth, 1 = Blood only, 2 = Urine only, 3 = Concordant, 4 = Discordant |
| Sex_Code | Binary | 0 = Female, 1 = Male |
| Medication_Amoxicillin | Binary | 1 if Amoxicillin is prescribed |
| Medication_Azithromycin | Binary | 1 if Azithromycin is prescribed |
| Medication_Ceftriaxone | Binary | 1 if Ceftriaxone is prescribed |
| Intensity_Score | Continuous | Squared value of Severity_Score to amplify the weighting of higher severities |
| Treatment Duration Clean | Continuous | Numeric extraction of days from treatment duration text (e.g., “10 days”) |
| Normalized_Duration | Continuous | Treatment duration divided by severity score |
| Resistance_Proxy_Score | Continuous | Proxy score: (1 – Culture_Concordance) × Normalized_Duration × Severity_Score |
| Hemoglobin_Status | Ordinal (0–2) | 1 = Anemic, 0 = Normal, 2 = High hemoglobin (sex-specific thresholds) |
| Platelet_Status | Ordinal (0–2) | 1 = Thrombocytopenia, 0 = Normal, 2 = Thrombocytosis |
| Calcium_Status | Ordinal (0–2) | 1 = Hypocalcemia, 0 = Normal, 2 = Hypercalcemia |
| Potassium_Status | Ordinal (0–2) | 1 = Hypokalemia, 0 = Normal, 2 = Hyperkalemia |
| Suspected_Typhoid | Binary | 1 if Salmonella present or (severity ≥ 2 and anemia or thrombocytopenia), 0 otherwise |
| SimMet_EnergyDisruption | Binary | 1 if hemoglobin is below the normal threshold (sex-specific), indicating ATP metabolism stress |
| SimMet_HypoxiaStress | Binary | Mirrored from SimMet_EnergyDisruption to indicate oxygen transport failure |
| SimMet_MembraneInstability | Binary | 1 if calcium <8.5 or >10.5 mg/dL, indicating disrupted membrane potential |
| SimMet_ElectrolyteDisruption | Binary | 1 if potassium is out of the 3.5–5.0 mmol/L range, reflecting acid–base imbalance |
| SimMet_CatabolicBurst | Binary | 1 if WBC >11×10⁹/L or severity ≥2, capturing catabolism under inflammatory stress |
| SimMet_InflammatoryStress | Binary | 1 if platelet count >450,000/µL, representing systemic inflammation |
| PC1 | Continuous | First principal component of standardized simulated metabolomic features |
| PC2 | Continuous | Second principal component of standardized simulated metabolomic features |


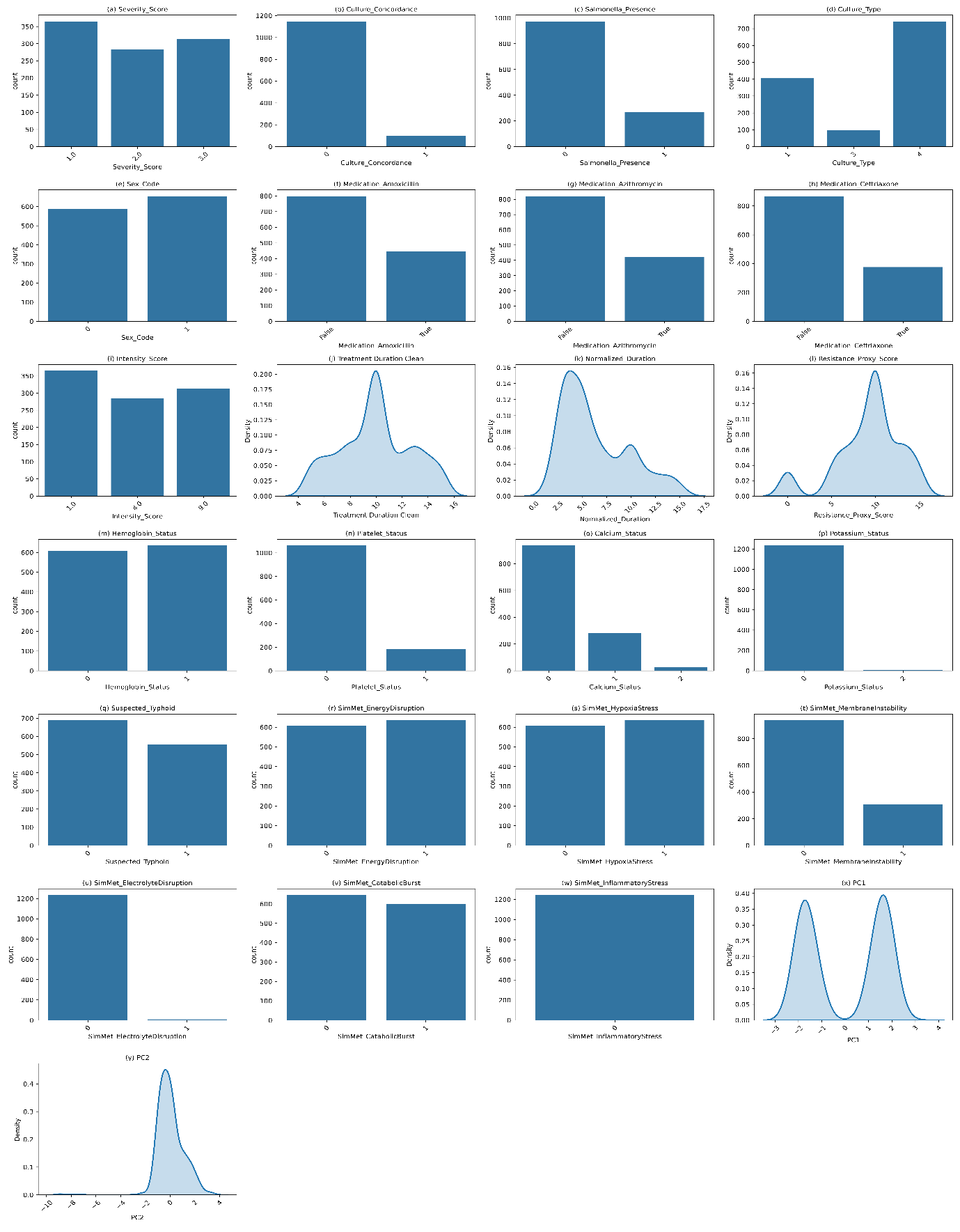


Figure S3: Distribution plots of engineered features following clinical transformation and simulated metabolomic augmentation. Binary and categorical features are shown using count plots, while continuous features are shown using kernel density estimates. The figure highlights balanced representation across clinical categories, non-trivial variance in biochemical features, and interpretable patterns in both rule-based and latent dimensions.


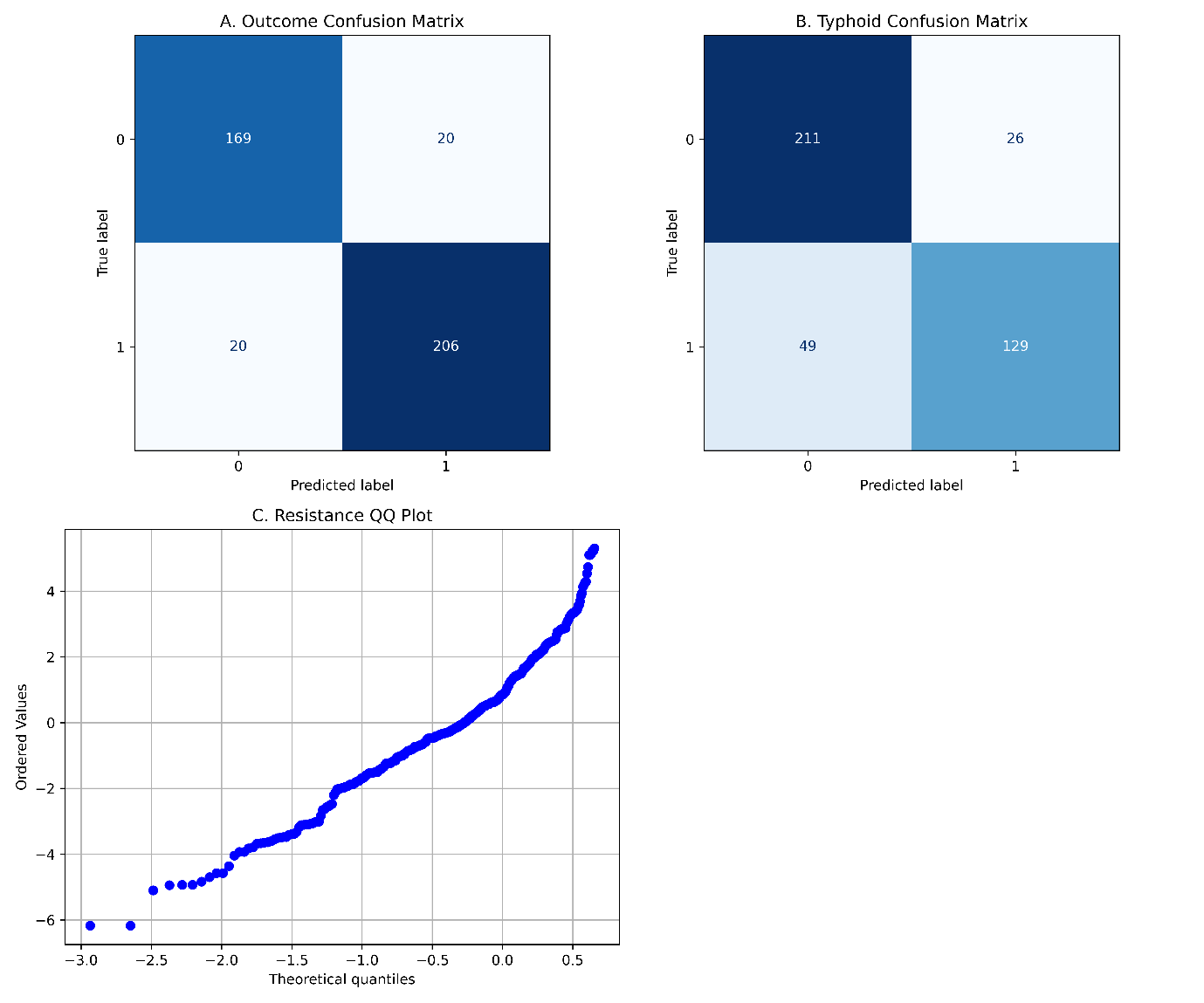


Figure S4: (A) and (B) present confusion matrices for the outcome and typhoid classifiers, confirming class-wise prediction accuracy. (C) shows a residual QQ plot for the resistance model.

Table S2: Top 20 ranked features by SHAP importance for each predictive model. Values represent the mean absolute SHAP value across test folds, indicating the relative contribution of each feature to model predictions.

| **Rank** | **Resistance Model** | **SHAP** | **Outcome Model** | **SHAP** | **Typhoid Model** | **SHAP** |
| --- | --- | --- | --- | --- | --- | --- |
| 1 | Culture_Type | 1.3845372 | Platelet Count | 0.7291237 | Severity_Score | 1.3532633 |
| 2 | Potassium (mmol/L) | 0.30348402 | Age | 0.7082083 | SimMet_EnergyDisruption | 0.95732945 |
| 3 | Hemoglobin (g/dL) | 0.2554697 | Calcium (mg/dL) | 0.49222493 | Platelet Count | 0.61405766 |
| 4 | Platelet Count | 0.23524752 | Potassium (mmol/L) | 0.47303808 | Age | 0.34936005 |
| 5 | Age | 0.19685143 | Hemoglobin (g/dL) | 0.4306213 | Hemoglobin (g/dL) | 0.3245283 |
| 6 | Calcium (mg/dL) | 0.15091972 | Medication_Amoxicillin | 0.25196788 | SimMet_CatabolicBurst | 0.32156345 |
| 7 | Medication_Ceftriaxone | 0.14120556 | Severity_Score | 0.21673025 | Resistance_Proxy_Score | 0.2908871 |
| 8 | Intensity_Score | 0.11367272 | Medication_Ceftriaxone | 0.16533513 | Normalized_Duration | 0.24966516 |
| 9 | Suspected_Typhoid | 0.07543601 | Suspected_Typhoid | 0.16077235 | SimMet_HypoxiaStress | 0.24574807 |
| 10 | SimMet_EnergyDisruption | 0.041111443 | Culture_Type | 0.15370034 | Potassium (mmol/L) | 0.23458262 |
| 11 | Medication_Amoxicillin | 0.038015716 | Sex_Code | 0.13996135 | Calcium (mg/dL) | 0.23076652 |
| 12 | Treatment Outcome | 0.03795603 | SimMet_CatabolicBurst | 0.120646335 | Culture_Concordance | 0.1832888 |
| 13 | Sex_Code | 0.033965014 | Medication_Azithromycin | 0.10567289 | Treatment Duration Clean | 0.13938992 |
| 14 | Medication_Azithromycin | 0.024217809 | SimMet_MembraneInstability | 0.07977929 | Culture_Type | 0.12543933 |
| 15 | Salmonella_Presence | 0.022132222 | Salmonella_Presence | 0.02956419 | Sex_Code | 0.07271386 |
| 16 | SimMet_MembraneInstability | 0.0073992372 | SimMet_EnergyDisruption | 0.016203111 | Treatment Outcome | 0.05744342 |
| 17 | SimMet_HypoxiaStress | 0.0 | Culture_Concordance | 0.014194644 | Platelet_Status | 0.05723731 |
| 18 | SimMet_ElectrolyteDisruption | 0.0 | Platelet_Status | 0.0 | SimMet_MembraneInstability | 0.020920184 |
| 19 | SimMet_CatabolicBurst | 0.0 | SimMet_HypoxiaStress | 0.0 | SimMet_ElectrolyteDisruption | 0.0 |
| 20 | SimMet_InflammatoryStress | 0.0 | SimMet_ElectrolyteDisruption | 0.0 | SimMet_InflammatoryStress | 0.0 |


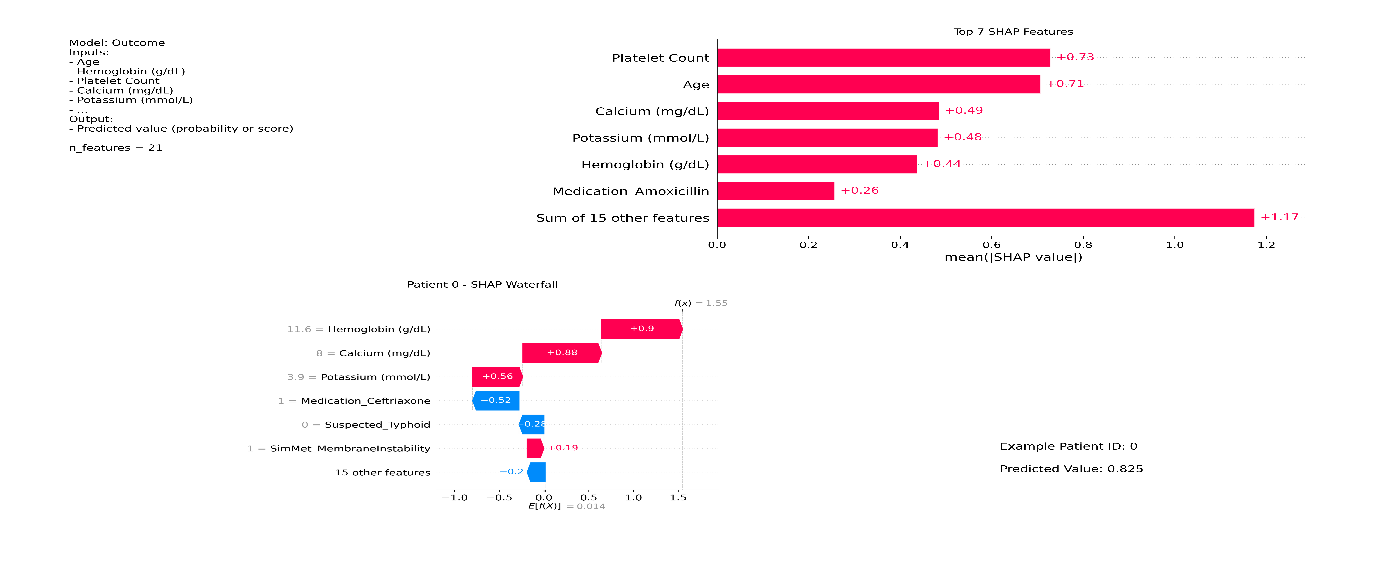


Figure S5: SHAP-based Classification Insight for treatment outcome model. Top patient attributed features SHAP features include platelet count, potassium, and age, suggesting key roles in binary discrimination. This waterfall plot illustrates the additive contributions of these features toward the predicted log-odds of typhoid classification for an individual case, supporting transparent clinical interpretation.


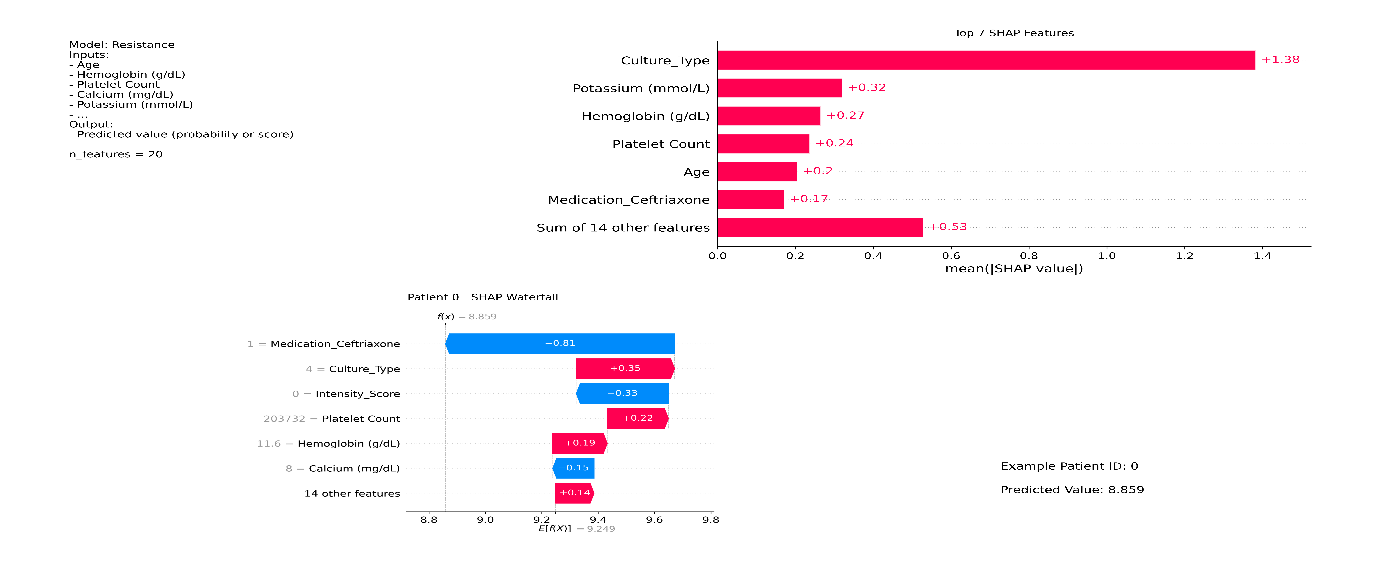


Figure S6: SHAP Feature Attributions for the regression model. The SHAP waterfall plot for a representative patient demonstrates how inputs cumulatively influence the resistance score prediction, offering insight into the model’s risk stratification.


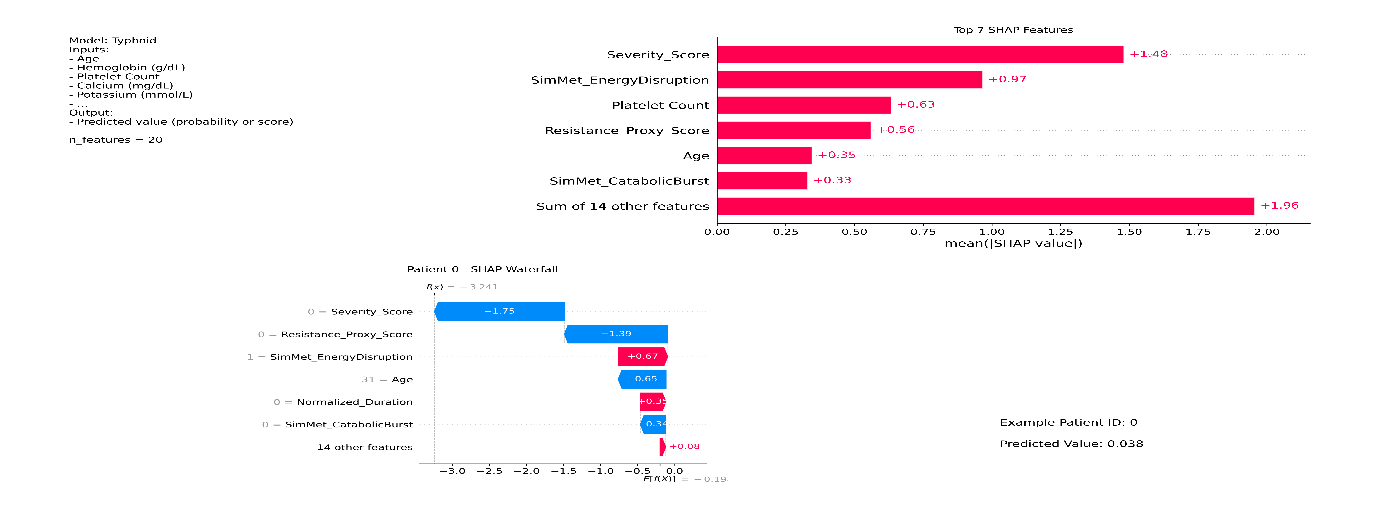


Figure S7: SHAP Interpretability of suspected typhoid. The individualized SHAP waterfall plot shows how specific features drive the model’s probability shift for a selected patient, enabling explainable prediction in complex, multivariate clinical settings.


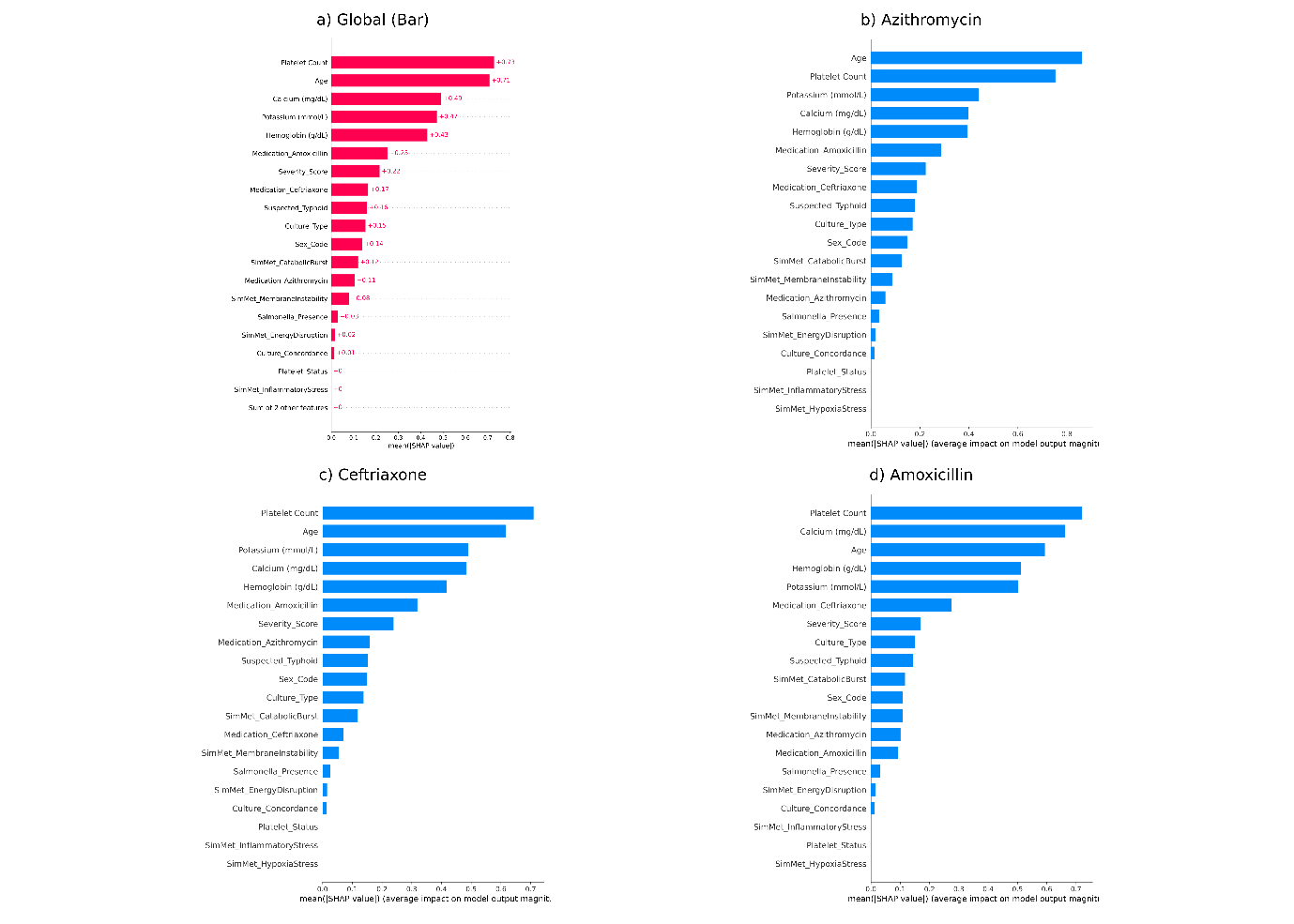


Figure S8: SHAP bar plots summarizing average feature importance globally (a) and by Top-1 predicted drug: Azithromycin (b), Ceftriaxone (c), and Amoxicillin (d). Feature rankings mirror those in the beeswarm plots.


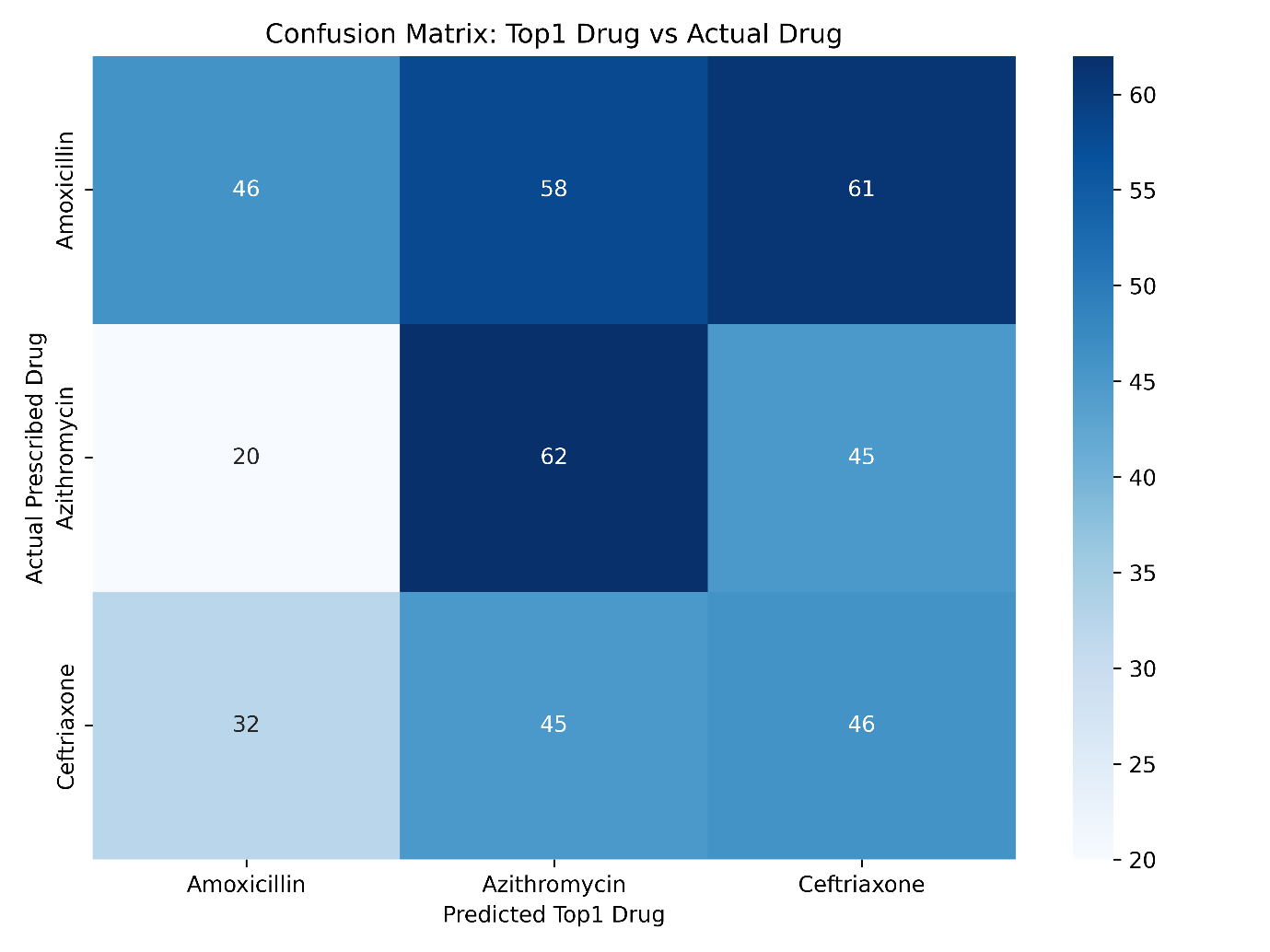


Figure S9: Confusion matrix for Top-1 predicted drugs versus actual prescriptions.

Table S3: Classification metrics for Top-1 predicted drugs compared to actual prescriptions.

| Drug | Precision | Recall | F1-Score | Support |
| --- | --- | --- | --- | --- |
| Amoxicillin | 0.47 | 0.28 | 0.35 | 165 |
| Azithromycin | 0.38 | 0.49 | 0.42 | 127 |
| Ceftriaxone | 0.30 | 0.37 | 0.33 | 123 |
| Accuracy |  |  | 0.3711 | 415 |
| Macro Avg | 0.38 | 0.38 | 0.37 | 415 |
| Weighted Avg | 0.39 | 0.37 | 0.37 | 415 |


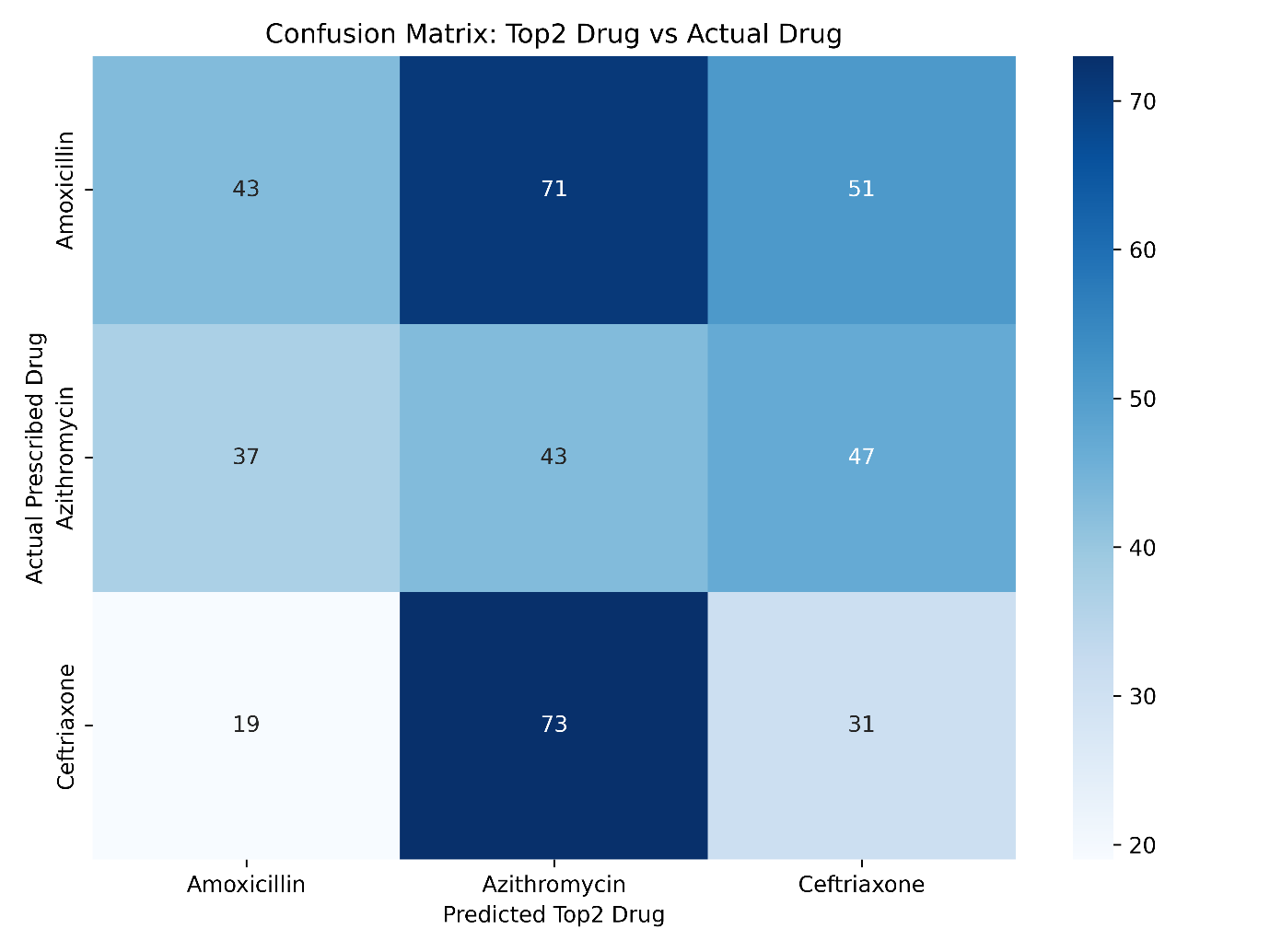


Figure S10: Confusion matrix for Top-2 predicted drugs versus actual prescriptions.

Table S4: Classification metrics for Top-2 predicted drugs compared to actual prescriptions.

| Drug | Precision | Recall | F1-Score | Support |
| --- | --- | --- | --- | --- |
| Amoxicillin | 0.43 | 0.26 | 0.33 | 165 |
| Azithromycin | 0.23 | 0.34 | 0.27 | 127 |
| Ceftriaxone | 0.24 | 0.25 | 0.25 | 123 |
| Accuracy |  |  | 0.2819 | 415 |
| Macro Avg | 0.30 | 0.28 | 0.28 | 415 |
| Weighted Avg | 0.31 | 0.28 | 0.29 | 415 |


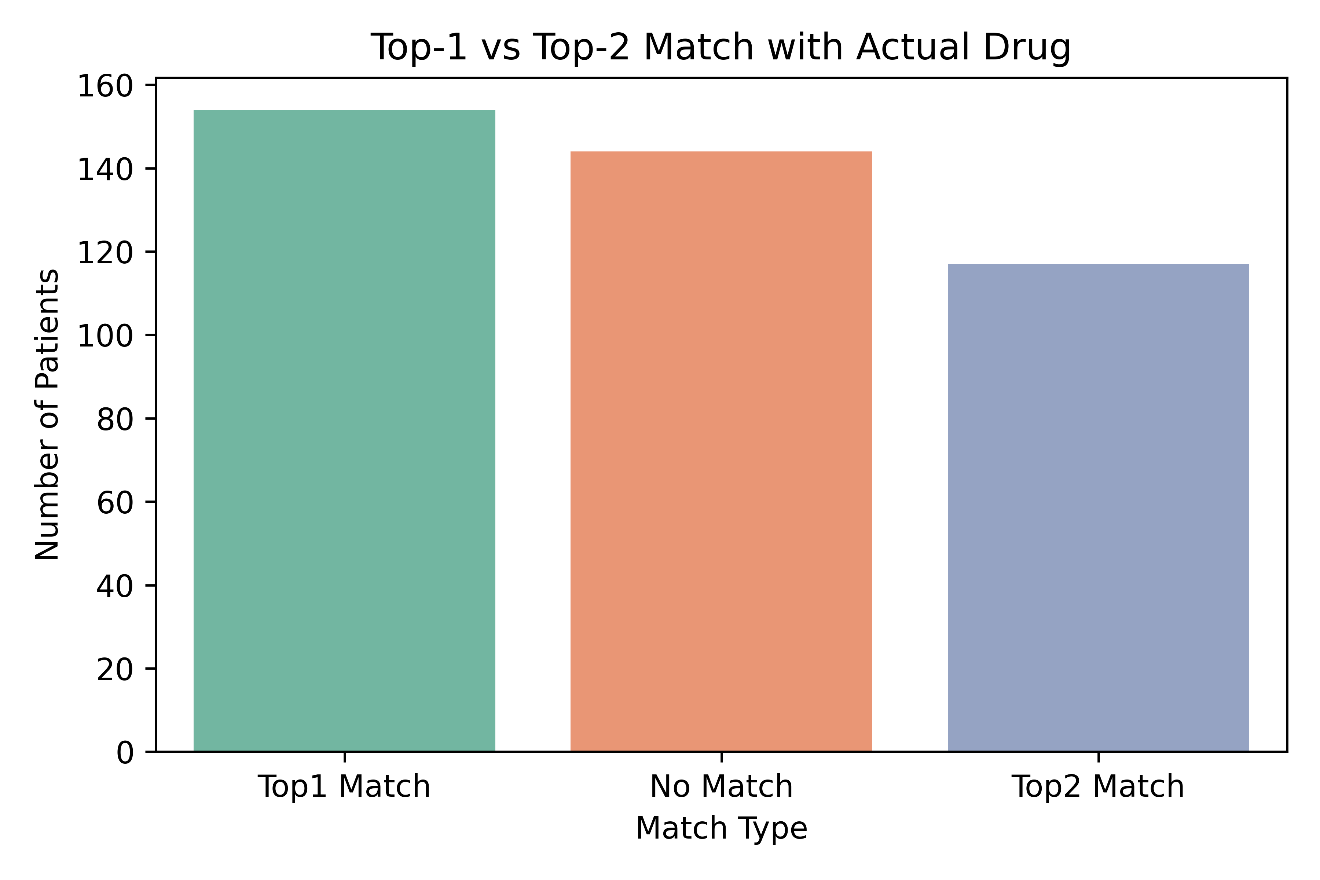


Figure S11: Top K analysis.

Table S5: Summary statistics of predicted treatment duration, predicted resistance proxy score, original treatment duration, and treatment outcomes stratified by match type. Top 1 matches show the highest alignment with successful treatment outcomes, while No Match cases correspond to poor outcomes.

| Match Type | Predicted Resistance Proxy Score Mean | Std | Count | Original Treatment Duration Mean | Std | Count | Treatment Outcome Mean | Std | Count |
| --- | --- | --- | --- | --- | --- | --- | --- | --- | --- |
| No Match | 8.63 | 3.68 | 144 | 9.92 | 2.78 | 144 | 0.33 | 0.47 | 144 |
| Top1 Match | 9.58 | 2.63 | 154 | 10.03 | 2.72 | 154 | 0.73 | 0.45 | 154 |
| Top2 Match | 9.32 | 2.80 | 117 | 10.00 | 2.62 | 117 | 0.57 | 0.50 | 117 |

Table S6: Summary of confidence classification outcomes for Top1 drug recommendations applied across 415 patient cases.

| Classification Type | Confident (n) | Ambiguous (n) | Confidence (%) |
| --- | --- | --- | --- |
| Absolute (≥ 0.5) | 242 | 173 | 58.31% |
| Relative (margin ≥ 0.01) | 302 | 113 | 72.77% |

Table S7: Summary of Treatment Discrepancy Outcomes. Breakdown of discrepant treatment cases, proportion of cases where the model outperformed the actual treatment, and high-confidence examples with Δ > 0.05. 10 Selected High-Confidence Discrepant Cases. Patients where the model recommendation differed from the actual drug prescribed, with Δ Probability > 0.05 and additional patient metadata for interpretability.

| Metric | Value |
| --- | --- |
| Total discrepant cases | 261 |
| The model outperformed the doctor | 261 (100.00%) |
| High-confidence examples (Δ > 0.05) | 10 |

Table S8: Stratified summary of treatment duration quartiles with corresponding resistance scores. Each quartile (Q1–Q4) contains 324 patients, ordered by increasing predicted treatment duration. Columns report the mean, minimum, and maximum duration per quartile, along with the mean resistance proxy score and patient count. Higher quartiles show a consistent increase in resistance risk, reflecting potentially more complex or resistant clinical cases.

| Risk Quartile | Mean Resistance Score | Min Score | Max Score | Patient Count |
| --- | --- | --- | --- | --- |
| Q1 (Lowest Risk) | 5.20 | −0.18 | 8.54 | 104 |
| Q2 | 9.27 | 8.55 | 9.83 | 104 |
| Q3 | 10.30 | 9.83 | 10.92 | 103 |
| Q4 (Highest Risk) | 11.96 | 10.96 | 14.04 | 104 |

Table S9: Performance summary by actual drug prescribed, showing patient count, mean Top-1 match probability, mean treatment duration, mean resistance proxy score, and treatment success rate. Azithromycin shows the highest success rate, while Ceftriaxone records the lowest among the three drugs analyzed.

| Actual_Drug | Patient_Count | Mean_Top1_Prob | Mean_Resistance_Proxy | Success_Rate |
| --- | --- | --- | --- | --- |
| Amoxicillin | 165 | 0.616 | 9.065 | 52.727 |
| Azithromycin | 127 | 0.622 | 9.509 | 62.992 |
| Ceftriaxone | 123 | 0.563 | 8.983 | 47.967 |
